# N-terminome analyses underscore the prevalence of SPPL3-mediated intramembrane proteolysis among Golgi-resident enzymes and its role in Golgi enzyme secretion

**DOI:** 10.1007/s00018-022-04163-y

**Published:** 2022-03-13

**Authors:** Laura Hobohm, Tomas Koudelka, Fenja H. Bahr, Jule Truberg, Sebastian Kapell, Sarah-Sophie Schacht, Daniel Meisinger, Marion Mengel, Alexander Jochimsen, Anna Hofmann, Lukas Heintz, Andreas Tholey, Matthias Voss

**Affiliations:** 1 Institute of Biochemistry, Kiel University, Rudolf-Höber-Str. 1, 24118 Kiel, Germany; 2 Systematic Proteome Research and Bioanalytics, Institute for Experimental Medicine, Kiel University, 24105 Kiel, Germany; 3 National Bioinformatics Infrastructure Sweden (NBIS), Science for Life Laboratory, Department of Biochemistry and Biophysics, Stockholm University, 10691 Stockholm, Sweden; 4 Present Address: Institute of Immunology, University Medical Center Schleswig-Holstein, 24105 Kiel, Germany; 5 Present Address: Institute for Cellular and Integrative Physiology, University Medical Center Hamburg Eppendorf, Hamburg, Germany

**Keywords:** SPPL3, Intramembrane proteolysis, Serum glycosyltransferases, GALNT2, FUT7, Extracellular glycosylation

## Abstract

Golgi membrane proteins such as glycosyltransferases and other glycan-modifying enzymes are key to glycosylation of proteins and lipids. Secretion of soluble Golgi enzymes that are released from their membrane anchor by endoprotease activity is a wide-spread yet largely unexplored phenomenon. The intramembrane protease SPPL3 can specifically cleave select Golgi enzymes, enabling their secretion and concomitantly altering global cellular glycosylation, yet the entire range of Golgi enzymes cleaved by SPPL3 under physiological conditions remains to be defined. Here, we established isogenic *SPPL3*-deficient HEK293 and HeLa cell lines and applied N-terminomics to identify substrates cleaved by SPPL3 and released into cell culture supernatants. With high confidence, our study identifies more than 20 substrates of SPPL3, including entirely novel substrates. Notably, our N-terminome analyses provide a comprehensive list of SPPL3 cleavage sites demonstrating that SPPL3-mediated shedding of Golgi enzymes occurs through intramembrane proteolysis. Through the use of chimeric glycosyltransferase constructs we show that transmembrane domains can determine cleavage by SPPL3. Using our cleavage site data, we surveyed public proteome data and found that SPPL3 cleavage products are present in human blood. We also generated HEK293 knock-in cells expressing the active site mutant D271A from the endogenous *SPPL3* locus. Immunoblot analyses revealed that secretion of select novel substrates such as the key mucin-type O-glycosylation enzyme GALNT2 is dependent on endogenous SPPL3 protease activity. In sum, our study expands the spectrum of known physiological substrates of SPPL3 corroborating its significant role in Golgi enzyme turnover and secretion as well as in the regulation of global glycosylation pathways.

**Supplementary Information:**

The online version contains supplementary material available at 10.1007/s00018-022-04163-y.

## Introduction

The Golgi apparatus is an integral part of the eukaryotic secretory pathway. Golgi cargo arrives from the endoplasmic reticulum (ER), is trafficked through the Golgi and eventually sorted into vesicles destined for the cell surface, the extracellular space or the endolysosomal compartment [2]. During Golgi transit, cargo proteins and lipids are subject to manifold modifications, among which glycosylation undoubtedly is particularly prominent [3]. The Golgi apparatus is central to synthesis of glycolipids and various types of *O*-glycans as well as the maturation of N-linked glycans on newly synthesized glycoproteins, because Golgi cisternae harbour a variety of enzymes that modify and diversify glycan structures in a complex, stepwise fashion. For the most part, these Golgi-resident glycan-modifying enzymes (GMEs), which include glycosyltransferases (GTfs), glycosidases and others, are membrane-anchored through an N-terminal transmembrane domain (TMD), i.e. adopt a type II topology [4].

A large fraction of Golgi GMEs is secreted into the extracellular space and can be detected in cell culture supernatants and body fluids [4–6]. While the intra-Golgi function of GMEs in glycan synthesis is evident, the (patho-)physiological relevance of Golgi GME secretion is incompletely understood [4, 7]. Given that GTf catalysis is strictly dependent on nucleoside-conjugated monosaccharide building blocks, which are scarcely available in the extracellular space, the prevailing hypothesis is that Golgi enzymes do not actively contribute to glycan synthesis or remodelling once secreted [7] making it conceivable that GTf secretion could rather serve the purpose of Golgi proteostasis. However, more recent observations corroborate enzyme-catalysed extracellular glycosylation in certain physiological settings and thus challenge the hypothesis that secreted Golgi GTfs do generally not display catalytic activity in the extracellular space. Upon activation, platelets, for example, can release soluble GTfs along with sugar nucleotides to fuel glycosylation [8–10] and a growing body of evidence supports a physiological role of extracellular sialylation in the hematopoietic system [11–13].

Given that the majority of Golgi GMEs are membrane-anchored, endoproteolysis is a prerequisite for their secretion. Even though proteolysis-dependent secretion is of such conceptual importance for Golgi enzyme secretion, the protease(s) catalysing GME cleavage and thus enabling secretion have long been elusive. BACE1 was reported to cleave ST6GAL1 in vitro [14] and in vivo [15] and to also cleave other sialyltransferases [16]. An independent line of work revealed that the intramembrane protease SPPL3 profoundly contributes to Golgi GME proteolysis. As member of the SPP/SPPL subfamily of GxGD-type aspartyl intramembrane proteases [17, 18] SPPL3 selectively cleaves type II membrane proteins within or very close to the exoplasmic boundary of the substrate’s TMD [17, 19, 20]. Notably, unlike other mammalian GxGD-type proteases, SPPL3 hydrolyses type II protein substrates without requiring prior cleavage by a distinct protease [21]. Candidate-based [19] and proteomic [20] approaches revealed that proteolysis-dependent secretion of select GTfs is affected by ectopic expression or loss of SPPL3. SPPL3 thus can control intra-Golgi levels of these enzymes explaining why forced changes in SPPL3 expression are associated with altered glycosylation patterns [19]. Importantly, the effect of SPPL3 is not limited to N-glycans as initially described (and later confirmed [22]), since subsequent unbiased genetic screens identified *SPPL3* as regulator of glycosphingolipid biosynthesis in the Golgi network [23–25] and since enzymes involved in various types of Golgi O-glycosylation were identified as SPPL3 substrates [20]. From a pathophysiological perspective an interesting role of SPPL3 was recently reported at the tumour-CD8^+^ T cell interface, since experimental loss of SPPL3 in tumour cells leads to diminished CD8^+^ T cell activation through a glycosphingolipid-dependent mechanism [24] and sub-clonal loss of SPPL3 in a variety of cancers appears to be associated with a less pronounced T cell response [26].

Thus, proteolytic cleavage of type II Golgi enzymes is a wide-spread phenomenon that has physiological implications on the cellular and organismal level, yet is incompletely understood. To further dissect specifically the role of SPPL3 in Golgi enzyme proteolysis and secretion, we applied N-terminome analyses to identify additional SPPL3 substrates and to obtain cleavage site information. To capture substrates of SPPL3 under steady-state physiological conditions, our study focusses on the use of isogenic knock-out cell models. We identified known but also novel SPPL3 substrates and provide a comprehensive list of likely physiological SPPL3 cleavage sites. Closer inspection of these revealed no overt SPPL3 consensus cleavage site but using chimeric constructs we found that the TMD of a prototypic SPPL3 substrate can determine SPPL3-mediated intramembrane proteolysis. Our study thus expands the spectrum of known physiological SPPL3 substrates which is going to be instrumental to further dissect the physiological function of SPPL3 in the Golgi and its applicability, for e.g. glycoediting, but also provides important mechanistic insights into SPPL3-mediated intramembrane proteolysis.

## Materials and methods

### Molecular cloning

Unless otherwise specified, directional cloning was performed following standard protocols and using molecular biology reagents from Thermo Fisher Scientific (Waltham, MA, USA). For the generation of transgene-expressing HEK293 Flp-In™ T-REx™ cells we first generated a compatible destination vector. Via oligo duplex annealing, we introduced *NheI* and *BshTI* sites into the MCS of pcDNA5/FRT/TO (Thermo Fisher Scientific) opened with *HindIII* and *XhoI*. Then, using *NheI* and *BshTI*, the attR1-CmR-ccdB-attR2 cassette was excised from pCW57.1 (#41393, Addgene, Watertown, MA, USA) and inserted into the pcDNA5 backbone to obtain pcDNA5/FRT/TO/DEST. SPPL3 CDS corresponding to GenBank RefSeq NM_139015.4 was kindly provided by R. Fluhrer (Augsburg, Germany) and was PCR-amplified using Q5 polymerase (New England Biolabs, Ipswich, MA, USA). *HindIII* and *XbaI* sites as well as the CDS for the C-terminal V5 or HA tags were introduced with the primers. Using *HindIII* and *XbaI*, SPPL3 CDS was transferred to pENTR1A-GFP-N2 (Addgene #19364) and subsequently shuttled to pcDNA5/FRT/TO/DEST using LR clonase II. For ectopic expression of C-terminally V5-tagged type II membrane proteins, we first introduced a V5 tag-encoding sequence followed by a stop codon and flanked by *BamHI* and *EcoRI* sites via oligo duplex annealing into pcDNA3.1(+) (Thermo Fisher Scientific). PCR-amplified GTf CDS was then cloned into this backbone in-frame with the V5 tag CDS via *HindIII* and *KpnI* or *BamHI.* FUT7 and MGAT5 cDNA (corresponding to NM_004479 and NM_002410, respectively) were kindly provided by Charles J. Dimitroff (Boston, USA) and R. Fluhrer (Augsburg, Germany), respectively. CHST14 and B3GAT3 were cloned from HME cell cDNA and correspond to Genbank entries NM_130468 and NM_012200, respectively. For the generation of a FUT7(TMD*)-V5 expression plasmid, FUT7 CDS was excised from pcDNA3.1 using *BamHI* and *HindIII* and transferred to a modified pmax plasmid backbone (kind gift of Yenan T. Bryceson, Stockholm, Sweden). This plasmid was subjected to PCR amplification to introduce *BsaI* cleavage sites flanking the TMD and enable seamless mutagenesis. Partially complementary oligonucleotides coding for the MGAT5 TMD were phosphorylated and annealed. The PCR-amplified plasmid and annealed oligo duplexes were mixed and digested with BsaI-HFv2 (New England Biolabs), followed by addition of T4 ligase (New England Biolabs) and *DpnI* (Thermo Fisher Scientific) and transformation into *E. coli* TOP10 (Thermo Fisher Scientific). FUT7 plasmids carrying the desired insertion were shuttled back to pcDNA3.1-V5 using *BamHI* and *HindIII*. All oligonucleotides were custom-made at Integrated DNA Technologies (Coralville, IA, USA) or Sigma–Aldrich and sequences are compiled in the Suppl. Tables. PCR conditions can be shared upon request. All plasmids generated and used in this study were validated by Sanger sequencing at Eurofins Genomics (Ebersberg, Germany).

### Cell lines, CRISPR/Cas9 genome editing and transfections

HEK293 and HeLa cells were obtained from ATCC (Manassas, VA, USA). All cells were cultivated under standard conditions in DMEM GlutaMAX™ medium (Thermo Fisher Scientific) supplemented with 10% fetal calf serum (PAN biotech, Aidenbach, Germany) and non-essential amino acids (Sigma–Aldrich, St. Luis, MO, USA) and confirmed negative for mycoplasma contamination by routine PCR. HEK293 Flp-In™ T-REx™ cells were from Thermo Fisher Scientific and were cultivated in the presence of 15 µg/ml blasticidin and 100 µg/ml zeocin (Invivogen, San Diego, CA, USA). Stably SPPL3-transfected HEK293 Flp-In™ T-Rex™ cells were generated according to the manufacturer’s instructions. In brief, parental cells were transfected with pOG44 and the SPPL3-encoding pcDNA5/FRT/TO plasmid at a ratio of 9:1 using Lipofectamin 2000 (Thermo Fisher Scientific) and 2 days after transfection the selection regimen was changed to 10 µg/ml blasticidin and 100 µg/ml hygromycin B gold (Invivogen). For SPPL3 overexpression experiments, culture medium was supplemented with 1 µg/ml doxycycline (Sigma–Aldrich). sgRNAs targeting SPPL3 exon 8 and the *TRAC* locus as well as recombinant *S. pyogenes* Cas9 2NLS were from Synthego (Redwood City, CA, USA). To generate isogenic *SPPL3* KO cells, Cas9 RNPs were prepared according to Synthego’s instructions. 1–2 × 10^5^ cells were electroporated using the Neon device (Thermo Fisher Scientific) with the following settings: 2 pulses, 1150 V and 20 ms each (HEK293 cells) and 2 pulses, 1005 V and 35 ms each (HeLa cells). Following electroporation, they were immediately plated in 12-well plates. Three days after electroporation, single-cell clones were established by limited dilution. Genotyping of on- and off-target sites was performed on extracted genomic DNA using DreamTaq PCR reagents (Thermo Fisher Scientific) and oligonucleotides listed in Suppl. Tables. Precise PCR conditions are available upon request. Sanger sequencing of gel-purified PCR products was performed at Eurofins Genomics and CLC Main Workbench 8 (Qiagen, Hilden, Germany) was used for analysis. SPPL3 D271A knock-in HEK293 cells were generated following electroporation with Cas9 RNPs containing a crRNA:tracrRNA duplex along with a ssODN (in this case all reagents were from Integrated DNA Technologies, Suppl. Tables). Single-cell clones were expanded and genotyped via Sanger sequencing. Plasmid DNA was transiently transfected using polyethylenimine (Polysciences, Warrington, PA, USA) according to standard protocols or TransIT-LT1 (Mirus Bio, Madison, WI, USA) according to the manufacturer’s instructions.

### Sample preparation, subcellular fractionation and immunoblotting

For experiments, cells were cultivated on poly-l-lysine-coated (Sigma–Aldrich) cell culture dishes. To examine SPPL3 substrate secretion, cells were washed with pre-warmed PBS and then kept in OptiMEM GlutaMax (Thermo Fisher Scientific) for 24–48 h. CM was collected and detached cells were removed by centrifugation at 500*g*. The supernatant was subjected to ultracentrifugation prior to analysis. Cells were washed with ice-cold PBS, harvested and lysed with ice-cold RIPA buffer (150 mM NaCl, 50 mM Tris HCL (pH 7.4), 1 mM EDTA, 0.5% (w/v) sodium deoxycholate, 0.1% (w/v) SDS, 1% (v/v) Triton X-100 and protease inhibitor mix (Roche, Basel, Switzerland)). For membrane isolation, cells were resuspended in ice-cold hypotonic buffer (10 mM Tris (pH 7.6), 1 mM EDTA, 1 mM EGTA, pH 7.6) and sheered using a 23G needle. Following sedimentation of nuclei (2400*g*, 5 min, 4 °C), membrane pellets were obtained by centrifugation at 21,000*g* or 100,000*g*. Membrane pellets were washed twice with carbonate puffer (0.1 M Na_2_CO_3_, 1 mM EDTA, pH 11.3), once with STE buffer (150 mM NaCl, 50 mM Tris, 2 mM EDTA, pH 7.6) and then lysed in RIPA. Total protein content was quantified using BCA or MicroBCA assay kits (Thermo Fisher Scientific). TCA precipitations of CM were done as described elsewhere [19]. Golgi enrichment was performed as described [27] with minor protocol modifications. In brief, harvested and PBS-washed cells were resuspended in homogenisation buffer [0.25 M sucrose, 10 mM Tris–HCl, 150 mM KCl, 1 mM MgCl_2_ and 2 × complete protease inhibitor mix without EDTA (Roche)] and homogenised in a Dounce homogenizer. Following centrifugation (800*g*, 10 min, 4 °C), the PNS was adjusted to 1.2 M sucrose and loaded on a discontinuous sucrose gradient (2 M, 1.3 M, 1.2 M, 1.0 M, 0.25 M sucrose in 10 mM Tris–HCl (bottom to top). The gradient was subjected to ultracentrifugation (100,000*g*, 3 h, 4 °C). Vesicles enriched at interphases were collected and sedimented by ultracentrifugation (100,000*g*, 30 min, 4 °C), washed with PBS and lysed in RIPA buffer with HEPES (150 mM NaCl, 10 mM HEPES (pH 7.4), 1 mM EDTA, 0.5% (w/v) sodium deoxycholate, 0.1% (w/v) SDS, 1% (v/v) Triton X-100). For gel electrophoresis sample buffer (4x: 424 mM Tris HCL, 546 mM Tris base, 8% (w/v) lithium dodecyl sulfate, 40% (v/v) glycerol, 2.04 mM EDTA, 0.8 mM SERVA Blue G250, 0.7 mM phenol red, pH 8.5) and DTT (final 50–100 mM) was added to all samples. Samples were run on self-cast Bis–Tris gels in 1 × MOPS buffer (50 mM MOPS, 50 mM Tris, 0.1% (w/v) SDS, 1 mM EDTA, pH 7.7) and subjected to colloidal Coomassie Brilliant Blue staining or tank blotting on PVDF membranes (Millipore) for 2 h at 90 V constant. For immunoblotting, antibodies were diluted in tris-buffered saline (20 mM Tris, 150 mM NaCl, pH 7.6) supplemented with 0.1% (v/v) Tween-20. The following primary antibodies were used: anti-SPPL3 (mouse mAb, clone 7F9), anti-V5 (rabbit pAb, #AB3792), anti-GALNT2 (rabbit pAb, #HPA011222; all from Sigma–Aldrich), anti-MGAT5 (mouse mAb, clone 706824), anti-B4GALT1 (goat pAb, #AF3609), anti-EXTL3 (goat pAb, #AF2635; all from R&D Systems, Minneapolis, MN, USA), anti-calnexin (rabbit pAb, #10427-2-AP), anti-MGAT1 (rabbit pAb, #15103-1-AP), anti-CHST3 (rabbit pAb, #18242-1-AP; all from Proteintech, Rosemont, IL, USA), anti-calnexin (mouse mAb, clone 35, Becton Dickinson, Franklin Lakes, NJ, USA), anti-HA (rat mAb, clone 3F10, Roche Diagnostics, Rotkreuz, Switzerland), anti-B3GAT3 (mouse mAb, clone D-7) and anti-POMK (mouse mAb, clone S-23; both from Santa Cruz Biotechnology, Dallas, TX, USA). A hybridoma supernatant with a previously described anti-GALNT2 mAb (clone UH2 6B7) [28] was kindly provided by Ulla Mandel and Henrik Clausen (Copenhagen, Denmark) and was diluted 1:1 with blocking reagent. HRP-conjugated secondary antibodies were from Dianova (Hamburg, Germany) or R&D Systems. Blots were developed using conventional enhanced chemiluminescence chemistry (Millipore) and signals were captured using a LAS4000 system (Fujifilm Life Sciences). Fluorescent signals from Coomassie Brilliant Blue G250 (CBB) total protein stains were acquired in the 700 nm channel of a Typhoon imager (Cytiva, Marlborough, MA, USA). Densitometric analyses were conducted using ImageQuant TL v8.2 (Cytiva), Adobe Photoshop was used for image processing and Prism (GraphPad, San Diego, CA, USA) for statistical analyses.

### Lectin flow cytometry

Confluent cells were detached from cell culture dishes by gentle resuspension in PBS and were transferred to V-bottom 96-well plates for staining (1–2 × 10^6^ cells/well). Prior to this, control cells were treated with 4 µg/ml kifunensine (Bio-Techne, Minneapolis, MN, USA) overnight. Cells were washed with staining buffer (PBS supplemented with 2% (v/v) fetal calf serum, 2 mM EDTA) and were then stained successively with a near-IR fluorescent reactive dye (1:500, Thermo Fisher Scientific), biotin-conjugated lectins and streptavidin-PE (1:1000, Thermo Fisher Scientific) diluted in FACS buffer. The following biotinylated lectins were used: *Sambucus nigra* (5 µg/ml), *Griffonia simplicifolia* (0.5 µg/ml), *Pisum sativum* agglutinin (1 µg/ml), *Lens culinaris* agglutinin (0.5 µg/ml), *Phaseolus vulgaris* erythroagglutinin (1 µg/ml), *Phaseolus vulgaris* leucoagglutinin (1 µg/ml), and succinylated wheat germ agglutinin (2 µg/ml) (all from Vector laboratories, San Francisco, CA, USA) as well as Concanavalin A Type IV (1 µg/ml) and *Tritium vulgaris* (0.5 µg/ml) (from Sigma–Aldrich). Stained cells were fixed with 2% formaldehyde (FA) in PBS (Polysciences) and were analysed using a FACSCanto II and the FlowJo 10.6.2 software package (Becton Dickinson).

### Immunofluorescence and confocal microscopy

Cells were seeded on poly-l-lysine-coated cover slips (thickness: 1.5H; Marienfeld, Lauda-Königshofen, Germany) in a 24 well plate (1–2 × 10^5^ cells/well) and transfected one day after seeding as detailed earlier. 24–48 h after transfection cells were washed with PBS and then fixed with 4% FA in PBS and permeabilized [0.2% (v/v) Triton X-100 and 50 mM NH_4_Cl in PBS] at RT for 20 min. Cells were blocked with PBS supplemented with 5% (v/v) normal goat serum (Thermo Fisher Scientific) and 1% (w/v) BSA (Aurion, Wageningen, Netherlands) at RT for 30 min and subsequently incubated overnight at 4 °C with primary antibodies diluted in the same buffer. The following antibodies were used: anti-V5 (1:200, mouse mAb, #46-0705, Thermo Fisher Scientific), anti-GM130 (1:2000, rabbit pAb, #12480S, Cell Signalling Technology, Denver, MA, USA) and anti-calnexin (1:500, rabbit pAb, #10427–2-AP, Proteintech). Following washing, cells were stained with the Alexa Fluor-conjugated secondary antibodies (1:500, Thermo Fisher Scientific) diluted in PBS supplemented with 5% (w/v) BSA for 1 h at RT and then mounted on glass slides (Marienfeld) using ProLong Glass Antifade Mountant (Thermo Fisher Scientific) and stored at 4 °C. Samples were imaged using a Zeiss LSM980 microscope (63 × objective) and images were processed with Image J Version 1.53c.

### Collection of conditioned medium samples for mass spectrometry and N-terminome analysis

Cells (three T175 flasks per sample) were grown to 70% confluency as detailed earlier and washed extensively with pre-warmed PBS before adding serum-free DMEM for 24 h. Culture medium was then changed to phenol red-free and serum-free DMEM and cells were incubated another 24 h. CM samples were collected and proteins were concentrated and changed to 100 mM HEPES, pH 7.0 using Millipore Amicon-Ultra-15 3 k concentrators. Protein content was quantified using a MicroBCA assay. With the expectation that SPPL3 overexpression boosts the release of substrates into the CM a different approach was undertaken for these samples and a total volume of 15 ml cell-free CM was freeze-dried without further concentration and buffer exchange.

### Label-free analysis of HEK293 cell samples

Volume of supernatant or Golgi-enriched fractions equal to 10 µg was used for label-free analysis. Samples were dried down under vacuum centrifugation and resuspended with 1% SDS with TEAB. A modified version of the Single-Pot Solid-Phase-enhanced Sample Preparation (SP3) protocol was used for sample processing [29]. Briefly, samples were reduced with Tris(2-carboxyethyl)phosphine hydrochloride (TCEP, 5 mM) and then alkylated with iodoacetamide (12.5 mM) before being quenched with DTT (15 mM). Samples were precipitated onto hydrophilic and hydrophobic Sera-Mag SpeedBeads (carboxylate-modified magnetic beads, GE Healthcare) using sixfold volume of ethanol. Samples were washed twice with 80% ethanol and then digested with trypsin (1:25, enzyme:protein) overnight at 37 °C in the presence of 100 mM TEAB. Peptides were removed from the beads with the aid of a magnet, acidified and then dried down and stored at −20 °C prior to analysis.

### N-terminomics

Supernatant samples were lyophilized, resuspended in 1% SDS and then cleaned via chloroform/methanol/water (CMW) precipitation to remove any interfering substances such as small primary amines which can impede labelling. Golgi samples were sonicated in the presence of 1% SDS and then CMW precipitated. Samples were resuspended in 6 M Guanidine × HCl in 100 mM TEAB and a BCA on a diluted fraction was performed to determine protein concentration. Approximately 100 µg of each sample were reduced with TCEP (5 mM final) for 30 min at 65 °C and then alkylated with iodoacetamide (12.5 mM final) at room temperature for 30 min. Samples were then labeled with TMT reagent (either TMT-6-plex or TMT-10-plex) in equal volume of DMSO so that the final concentration of DMSO was 50%. The samples were left to react for 1 h at 25 °C and then quenched with hydroxylamine (1% final) for 30 min at 37 °C. All channels were combined and the sample was CMW precipitated. The pellet was washed with methanol, redissolved in 3 M Guanidine HCl and diluted to < 0.85 M with 100 mM TEAB. Trypsin was added (40:1, protein to enzyme) and the sample left to digest overnight at 37 °C. The sample was cleaned using a C_18_ column (50 mg, 1 cc, SepPak, Waters, Milford, MA, USA) and eluted with 80% acetonitrile (ACN) in 0.1% trifluoroacetic acid (TFA). One tenth or one twentieth (approximately 30 µg) of the sample was set aside for “Pre-HYTANE” while the rest of the sample was taken and the neo-N-termini generated as a result of trypsin digestion were depleted using HYTANE [30]. Samples were redissolved in HEPES buffer (pH 7.0) and then hexadecanal (500 µl, 10 mg/ml) in isopropanol was added along with 20 mM sodium cyanoborohydride. The reaction was left for ca. 4 h at 50 °C, followed by addition of sodium cyanoborohydride (20 mM). Samples were dried down at 60 °C for ca. 2 h using a vacuum centrifuge, acidified (1% TFA) and then brought to 1 ml in loading buffer (3% ACN, 0.1% TFA). Samples were spun down (10 min @ 20,000*g*) and cleaned with a C_18_ column. The sample was dried (vacuum evaporation) and stored at − 20 °C prior to analysis. If sample amounts permitted (> 600 µg after combining TMT channels), a portion of the HYTANE sample (85%) was also used for 2D N-terminomics. Here, samples were pre-fractionated over a 300 µm × 250 mm capillary column (C_18_, 110 Å, 5 µm particle size) using a gradient of 0.1% TFA in water (buffer A) and 0.1%TFA in ACN (buffer B) and a flow rate of 6 µL/min. Samples were separated over 60 min from 3 to 40% ACN, collected every minute and concatenated to six fractions. Samples were dried down and resuspended prior to LC–MS analysis.

### LC/MS measurements

All samples were injected in duplicate, except for 2D-LC which were measured only once. Samples were analyzed on a Dionex Ultimate 3000 nano-UHPLC coupled to either a Q Exactive HF mass spectrometer, QExactive Plus or an Orbitrap Fusion Lumos (all Thermo Fisher Scientific). The samples were washed on a trap column (Acclaim Pepmap 100 C_18_, 5 mm × 300 μm, 5 μm, 100 Å, Thermo Fisher Scientific) for 4 min with 3% ACN/0.1% TFA at a flow rate of 30 μl/min prior to peptide separation using an Acclaim PepMap 100 C_18_ analytical column (50 cm × 75 μm, 2 μm, 100 Å). A flow rate of 300 nl/min using eluent A (0.05% FA) and eluent B (80% ACN/0.04% FA) was used for gradient separation (120 min for 2D-LC and 180 min for 1D-LC, 5–40% B). Spray voltage applied on a metal-coated PicoTip emitter (10 μm tip size, New Objective, Woburn, MA, USA) was 1.7–1.9 kV, with a source temperature of 250 °C. MS and MS/MS parameters varied slightly between machines and between label-free and TMT experiments (TMT-6-plex and TMT-10-plex). Label-free experiments on HEK293 cells were measured on the QExactive HF with the following parameters: Full scan MS spectra were acquired between 375 and 1400 m/z at a resolution of 60,000 at m/z 200 and the top ten most intense precursors with charge states greater than 2 + were selected for fragmentation using an isolation window of 1.4 m/z and with HCD normalized collision energies (NCE) of 28 at a resolution of 30,000. Lock mass (445.120025) and dynamic exclusion (30 s) were enabled. For TMT experiments measured on the Fusion Lumos, full scan MS spectra were acquired between 375 and 1400 m/z at a resolution of 60,000 at m/z 200 and the most intense precursors with charge states greater than 2 + were selected for fragmentation for 3 s with an isolation window of 1.4 m/z and with HCD NCE of 38 at a resolution of 30,000. Lock mass (445.120025) and dynamic exclusion (30 s) were enabled. FAIMS was also implemented for 1D experiments. Here a 1 s cycle time was used for three different compensation voltages, i.e. (− 45, − 60 and − 75) or (− 50, − 65 and − 85). Technical replicates had different compensation voltages to increase the number of peptide identifications. For the 1D and 2D-LC HYTANE experiment the samples were measured on the Q Exactive HF. Full MS spectra were acquired between 375 and 1400 m/z at a resolution of 70,000 at m/z 200 and the ten most intense precursors with charge states greater than 2 + were selected for fragmentation with an isolation window of 1.2 m/z and with HCD NCE of 33 at a resolution of 30,000. Dynamic exclusion was set to 20 s. LC HYTANE measurements on the Q Exactive Plus (Flp-In™ T-REx™ HEK293 cells overexpressing SPPL3) full scan MS spectra were acquired between 300 and 2000 m/z at a resolution of 70,000 at m/z 200 and the ten most intense precursors with charge states greater than 2 + were selected for fragmentation using an isolation window of 1.4 m/z and with HCD NCE of 33 at a resolution of 17,500. For HeLa cells, a TMT-10 plex kit (9 from 10 channels) was used for labelling. As such higher resolution was required for MS2 experiments, while MS1 parameters were the same as above. Samples were measured (2D-LC) on both the Q Exactive HF and the Orbitrap Fusion Lumos. On the Orbitrap Lumos, 60,000 resolution was used with a maximum injection time of 118 ms and an NCE of 40. On the Q Exactive HF the resolution was also increased to 60,000 and the maximum injection time increased from 100 to 120 ms. All LC–MS data have been deposited to the ProteomeXchange Consortium via the PRIDE partner repository [1] with the dataset identifier PXD028769.

### Database search and statistics

The MS raw files were processed by Proteome Discoverer 2.2 and MS/MS spectra were searched using the Sequest HT algorithm against a database containing common contaminants and a canonical human database. For label-free experiments, samples were searched with two search nodes: utilizing both tryptic and semi-tryptic enzyme specificity, both with two missed cleavages allowed. An MS1 tolerance of 10 ppm and a MS2 tolerance of 0.02 Da was implemented. Oxidation (15.995 Da) of Met residues was set as a variable modification while carbamidomethylation (57.02146 Da) on Cys was set as a static modification. In addition, acetylation (42.011 Da), loss of Met (− 131.040 Da) and loss of Met and acetylation (− 89.030 Da) at the protein level was also set as a variable modification. Minimal peptide length was set to 7 aa and the peptide false discovery rate (FDR) was set to 1%. Label-free quantification was performed using the Minora feature detection. Normalized protein abundances from Proteome Discoverer were exported. Technical injections were averaged, the data were log 2 transformed, filtered so that at each protein had to be present in at least two out of three biological replicates in one of the samples (WT or KO). Missing values were imputed in Perseus (Perseus_1.6.10.43). Imputed values were randomly drawn from a normal distribution with a width of 0.3 and down shifted by 1.8 standard deviations. Statistical analysis (*t* test) was performed in Excel (two-tailed, unequal variance). For TMT experiments enzyme specificity was set to semi-ArgC with two missed cleavages allowed. An MS1 tolerance of 10 ppm and a MS2 tolerance of 0.02 Da was implemented. Oxidation (15.995 Da) of Met residues, acetylation (42.011 Da) and TMT-6-plex/TMT-10-plex (229.163 Da) on the peptide N-terminus was set as a variable modification while carbamidomethylation (57.02146 Da) on Cys residues and TMT on Lys residues was set as a static modification. Technical injection replicates were set as fractions. Minimal peptide length was set to six amino acids and the FDR was set to 1%. Normalized, scaled abundance from Proteome Discoverer were exported, log2-transformed and statistical analysis (*t* test, standard student’s *t* test assuming equal variance) were performed in Perseus (Perseus_1.6.10.43). In the N-terminomics data for HEK 293 cells (Fig. 2c, d), sample from KO clone #1 was considerably different from samples from clones #5 and #6, and thus omitted in *p* value calculation, log2 fold-change difference and SD calculations used to determine fold-change cut-offs. However, all the data are in supplied Suppl. Tables. Permutation-based FDR calculation on the remaining HEK dataset was performed in Perseus to get an FDR-adjusted *p* value (*q* value). Along with *SPPL3*-deficient HeLa cells clones and parental HeLa cells we also included a HeLa cell pool in which the *TRAC* locus was targeted by Cas9 as control. As we did not detect differences between parental and *TRAC*-targeted HeLa cells in respect to type II membrane protein secretion, the latter have been omitted from the manuscript.

### Data analysis, visualisation and peptide motif searches

Volcano plots were generated using the ggplot2 package in R. For protein and peptide filtering, protein topology information was retrieved from the Uniprot human reference proteome (current as of January 2021). Proteins were grouped as type II membrane protein when annotated as “single-pass type II membrane protein” (parameter: subcellular location) or carrying a “signal-anchor for type II membrane protein” (parameter: transmembrane). PSSMs were calculated using PSSMSearch [31] (set to Homo sapiens, method log odds, otherwise standard settings were used). For SPPL3 substrates, aa sequences flanking the scissile bond (P15 to P3’) were extracted. As control, an identical number of proteins annotated in Uniprot as type II membrane proteins displaying Golgi localisation and excluding known SPPL3 substrates were randomly selected and peptides spanning the last 18 aa of the annotated TMDs were extracted using Python. Sequence logo plots were generated from the calculated PSSMs using the Python package logomaker. R and Python code can be made available upon request. IceLogo analysis was performed using the IceLogo server [32].

## Results

### Identification of SPPL3 substrates in conditioned supernatants of isogenic knock-out (KO) HEK293 cell lines using N-terminomics

**Fig. 1:**
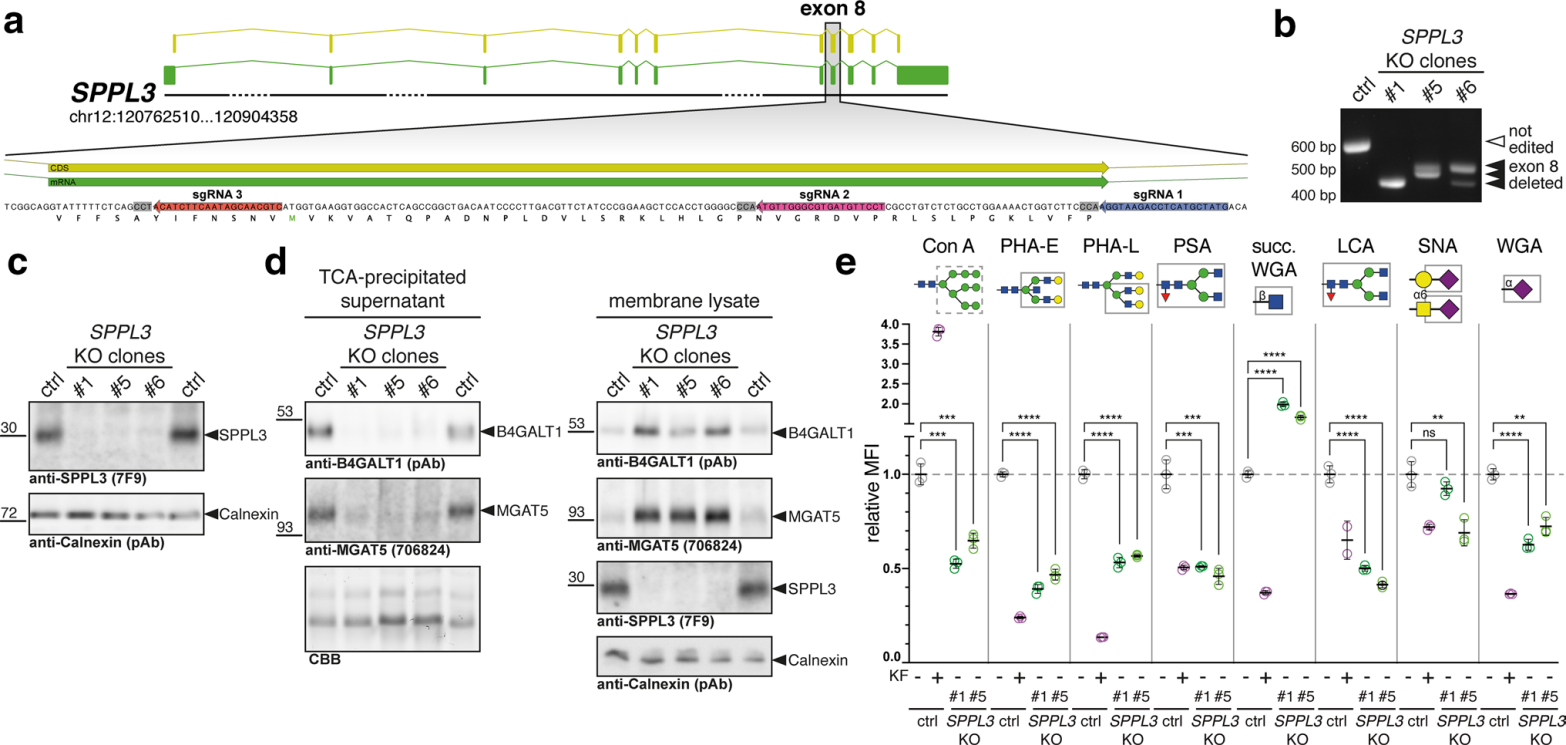
Generation of isogenic SPPL3-deficient HEK293 cell lines using CRISPR/Cas9 genome editing. **a** Schematic overview of the targeting strategy. To disrupt *SPPL3*, three distinct sgRNAs were used simultaneously that target exon 8 of the *SPPL3* locus. Coloured arrows indicate sgRNA binding sites; grey background highlights NGG PAM sites. Green annotations, exonic regions; yellow annotations, protein-coding regions. **b** PCR genotyping of obtained HEK293 cell clones. gDNA was extracted and a DNA fragment comprising exon 8 was amplified by PCR using specific primers binding in the flanking introns. In parental HEK293 cells (ctrl), an expected 545 bp band was amplified, while shorter bands observed in edited clones point to exon 8 deletions. **c** SPPL3 immunoblotting. Lysates of carbonate-washed cellular membranes were probed for endogenous SPPL3; calnexin was used as a loading control. **d** Detection of MGAT5 and B4GALT1 in culture supernatants and cellular membranes from edited cells and parental HEK293 cells using antibodies directed against the luminal domains of these GTfs. TCA-precipitated conditioned supernatants and carbonate-washed cellular membranes were subjected to immunoblotting with the indicated antibodies. Calnexin was used as a loading control for the membrane fraction, while total protein staining with colloidal CBB served as loading control for the conditioned supernatant. **e** Lectin flow cytometry. Cells were harvested and stained with the indicated biotinylated lectins as well as a streptavidin-PE conjugate. Data shown are obtained following singlet gating and exclusion of dead-cell marker-positive cells. Mean fluorescence intensity (MFI) was normalized to lectin-stained parental HEK293 cells (ctrl). To control for glycan-specific staining, kifunensine-treated cells (KF, 4 µg/ml overnight) were included in the experiment. Bar, mean ± SD

To identify novel SPPL3 substrates and obtain cleavage site information with terminomics we generated isogenic *SPPL3*-deficient clonal HEK293 cell lines using a multi-guide approach targeting exon 8 of the *SPPL3* locus on human chromosome 12 (Fig. 1a). HEK293 cells were simultaneously electroporated with Cas9 RNPs containing three distinct sgRNAs and 72 h after electroporation cells were singularized and expanded. PCR amplification of *SPPL3* exon 8 from genomic DNA (gDNA) of successfully edited clones revealed a complete loss of the 545 bp band present in parental HEK293 cells. Instead, smaller fragments were amplified exclusively, indicating that parts of exon 8 of *SPPL3* had been deleted following Cas9 cleavage at two sgRNA binding sites (Fig. 1b), which could also be corroborated by Sanger sequencing of purified PCR products (Suppl. Figure 1a). To ultimately demonstrate loss of SPPL3 expression at the protein level, we performed immunoblotting using a previously described anti-SPPL3 mAb (clone 7F9) [21]. As evident from Fig. 1c, endogenous SPPL3, which was readily detected in membranes isolated from parental HEK293 cells, was completely lost in Cas9-edited clones. We also did not detect any truncated SPPL3 fragments in these cells (data not shown). As off-target editing is a known draw-back of genome editing, we designed specific primers to amplify genomic regions predicted to constitute potential off-target binding sites of the sgRNAs used (Suppl. Figure 2). Sanger sequencing of the isogenic *SPPL3*-deficient clones established here did not reveal evidence of off-target editing at any of the predicted sites, which notably also includes a *SPPL3* pseudogene on chromosome 18. Thus, we can rule out wide-spread off-target edits in the clonal cell lines generated. Finally, we examined whether the loss of SPPL3 expression is associated with any of the previously described phenotypic glycosylation changes [19]. As expected, levels of the GTfs MGAT5 and B4GALT1, two SPPL3 substrates reported before [19], were profoundly reduced in the conditioned supernatants of the *SPPL3*-deficient cells and accumulated in the membrane fraction, which is consistent with impaired proteolysis and secretion due to the loss of SPPL3 (Fig. 1d). (Note that, for the purpose of clarity, we will use gene names to refer to SPPL3 substrates throughout this manuscript.) Such intracellular accumulation of excess active GTfs had previously been associated with a glycosylation phenotype [19]. We thus profiled the surface glycome of the *SPPL3*-deficient cell clones along with parental HEK293 cells using lectin flow cytometry (Fig. 1e). Cells treated with kifunensine, an α-mannosidase inhibitor known to halt complex *N*-glycosylation [33], were analysed in parallel and displayed markedly changed reactivity for concanavalin A and other lectins binding prominent *N*-glycan motifs, demonstrating that the lectin staining performed was glycan-specific. *SPPL3*-deficient clones displayed a robust and consistent glycosylation phenotype as evidenced by significantly altered staining with concanavalin A, PHA-E, PSA and ECL. Most notably, loss of SPPL3 led to a substantially increased binding of succinylated wheat germ agglutinin (WGA), which contrasts with the markedly reduced staining observed for most other lectins displaying an altered surface binding. Given that MGAT5, which produces the β1,6-branched N-glycans bound by PHA-L, is a known SPPL3 substrate that accumulates in *SPPL3*-deficient cells [19] (Fig. 1d), the reduction of PHA-L staining in *SPPL3*-deficient clones was unexpected yet is in line with observations made in *Sppl3*-deficient murine natural killer cells in vivo [34]. Taken together, we successfully established and characterized isogenic *SPPL3* KO HEK293 clones that represent a valid model for the identification of additional SPPL3 substrates.

**Fig. 2:**
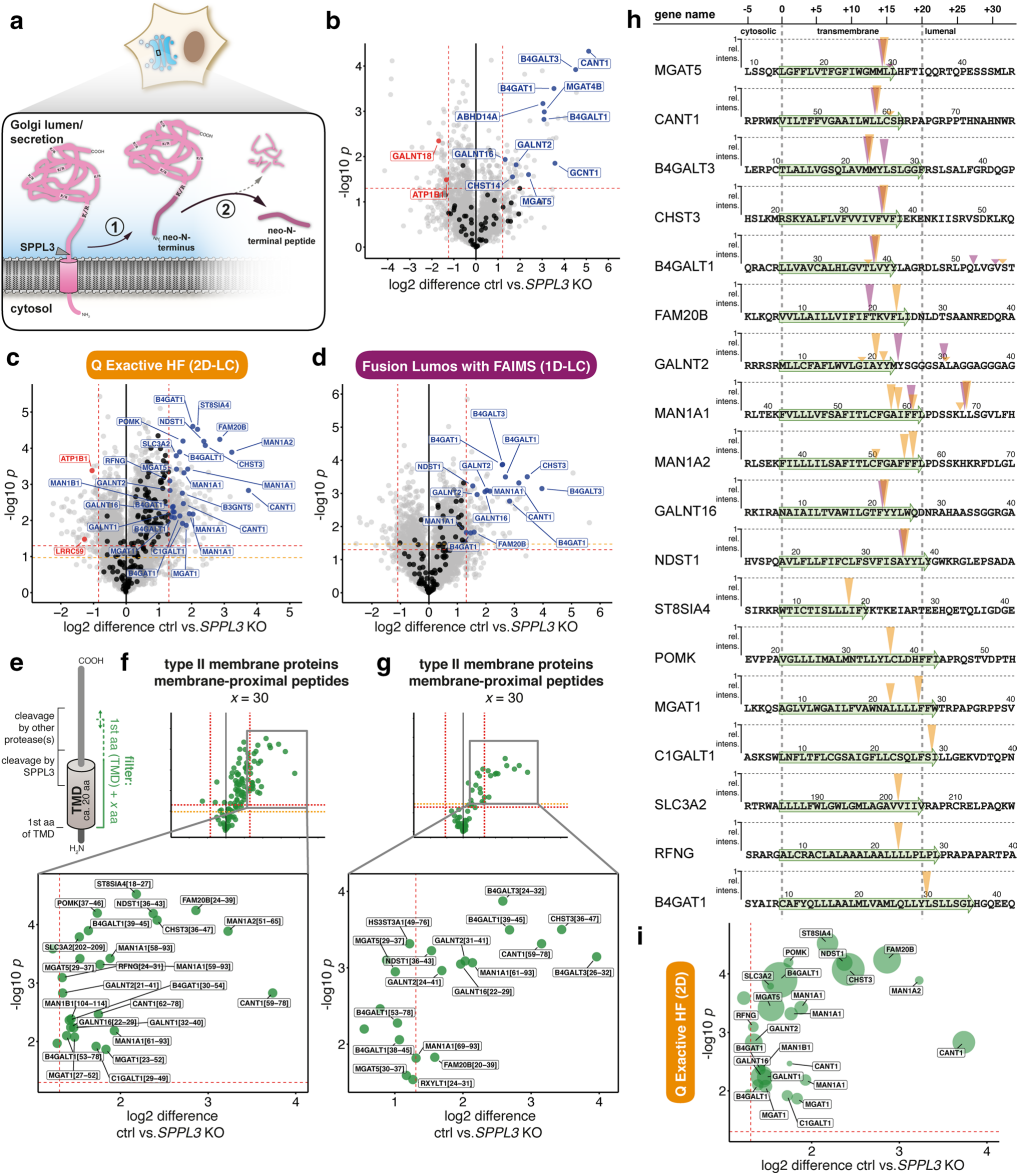
N-terminome enrichment approach to identify novel substrates of endogenous SPPL3 and their cleavage sites in CM of isogenic *SPPL3*-deficient HEK293 cell lines. **a** Schematic overview of SPPL3-mediated substrate cleavage in the Golgi (blue) and the experimental strategy followed here. Type II membrane protein substrates (pink, with internal trypsin cleavage sites (K/R)) are cleaved by SPPL3 (or other proteases) in the Golgi, are thus liberated from their membrane anchor and secreted (**1**). Due to proteolysis, liberated cleavage products have free N-terminal amino groups which can be selectively analysed following negative enrichment enabling N-terminome analysis (**2**). **b** Label-free quantification of serum-free CM of the three *SPPL3*-deficient isogenic HEK293 clones and parental HEK293 cells. A total of 3,459 proteins were identified and 131 had their missing values imputed. Proteins derived from type II membrane proteins (based on Uniprot annotations) are displayed in black as well as red or blue, if significantly more abundant in CM of *SPPL3* KO or parental cells, respectively. All other proteins identified are given in grey. Red dashed lines represent thresholds applied (log2 difference: median ± 2 SD; *p* = 0.05 (Welch's *t* test, two-tail, unequal variance). Gene names are used for labelling. **c**, **d** Volcano plots depicting changes in neo-N-terminal peptides enriched via HYTANE from CM of *SPPL3*-deficient (clone #5 and #6) as well parental cells as measured on the Q Exactive HF system (**c**, 9,477 unique peptides) and the Fusion Lumos with FAIMS (**d** 4628 unique peptides). Dashed lines indicate applied thresholds [log2 difference: median ± 2 SD; *p* = 0.05 (red) and FDR-adjusted *p* value (orange)]. Volcano plots are filtered for peptides from type II membrane proteins and labelled as in **b**. **e** Rationale of peptide filtering. GTfs and other Golgi enzymes adopt a type II membrane topology and their active site containing-ectodomains are anchored to the membrane through a stem region and an N-terminal TMD. Proteolytic cleavage is known to occur in these regions. SPPL3 is expected to cleave type II substrates in the exoplasmic half of the annotated TMD or in a luminal stretch very close to the annotated TMD, other proteases may cleave more distantly from the TMD. Peptides with an N-terminal residue originating in a window comprising the 1st TMD residue + *x* aa are given in green in subsequent volcano plots. **f**, **g** HYTANE datasets shown in **c**, **d** but filtered for peptides generated by membrane-proximal cleavage of type II membrane proteins (green). Peptides are labelled with gene name and peptide position (1st aa-last aa). **h** Schematic mapping of type II membrane protein-derived, TMD-proximal peptides reduced in the CM of *SPPL3* KO cells. Protein sequences comprising the TMD and flanking regions were retrieved from Uniprot and aligned at the annotated 1st aa of the TMD (green). Arrowheads indicate cleavage sites (yellow: data from **c**, purple from **d**). In the event that multiple membrane-proximal peptides were identified for one protein, height of arrowhead corresponds to the relative intensity of these peptides (mean of three HEK293 replicates). **i** Data from **c**, but with point size specifying measured relative peptide intensities (mean of three HEK293 replicates)

SPPL3 cleaves full-length type II membrane proteins [19, 20, 24], likely in the Golgi network, and substrate cleavage gives rise to soluble proteoforms with neo-N-termini, which are subsequently secreted (Fig. 2a). Such neo-N-termini can be identified using N-terminomics [35]. To this end, we performed negative enrichment via HYTANE [30] on serum-free conditioned media collected from *SPPL3* KO and parental HEK293 cells to selectively capture neo-N-termini and to identify substrates of endogenous SPPL3. Following labelling yet prior to enrichment, we first examined protein abundance in CM through quantification of peptides containing TMT-labelled Lys residues (Suppl. Figure 3a). This, however, only led to quantification of a limited number of type II membrane proteins. In contrast, using label-free quantification we found that eleven type II membrane proteins were depleted from CM of *SPPL3* KO cells (Fig. 2b). This included previously described substrates of SPPL3 (MGAT5, CANT1, B4GAT1 and B4GALT1), confirming the validity of our approach, but also novel candidate substrates, for instance GALNT2. Following enrichment of neo-N-terminal peptides via HYTANE, data were acquired on two distinct LC–MS pipelines, Q Exactive HF (Fig. 2c) and Fusion Lumos with FAIMS (Fig. 2d). Substantially more peptides were identified using the former, but in both datasets the majority of mapped peptides were derived from protein N-termini demonstrating successful enrichment via HYTANE (Suppl. Figure 3b). As SPPL3 is expected to only cleave type II membrane proteins [17, 19–21], we filtered both datasets accordingly and found that a substantial fraction of type II membrane protein-derived peptides were depleted from CM of *SPPL3*-deficient cells, suggesting these peptides are secreted in a SPPL3-dependent manner and could derive from direct cleavage catalysed by SPPL3 (Fig. 2c, d). With very high stringency, we first considered peptides with a log2-fold change > median ± 2 SD. Several such peptides surpassed the thresholds set and mapped chiefly to enzymes implicated in cellular glycosylation pathways. To specifically identify those peptides generated directly by SPPL3 cleavage, we next filtered for peptides that stem from a narrow 30-aa region comprising the annotated TMD and directly adjacent luminal aa to correct for imprecise TMD predictions (Fig. 2e). Cleavage within this region would be compatible with SPPL3-mediated intramembrane proteolysis, whereas other proteases such as BACE1 would most likely cleave at later positions in the stem region. Applying this filtering, we found that the majority of type II membrane protein-derived peptides more abundant in CM of parental cells did in fact originate within annotated TMDs, suggesting they are likely liberated by intramembrane proteolysis (Fig. 2f, g). In most cases, both datasets include peptides pinpointing identical cleavage sites, only for FAM20B, GALNT2 and MAN1A1 distinct but closely adjacent cleavage sites in the TMDs were identified (Fig. 2h). Whenever we identified multiple filtering criteria-matching peptides secreted in a SPPL3-dependent fashion, we calculated relative peptide intensities to identify those cleavage products most abundant in HEK293 cell CM, though this analysis may be biased by peptide-specific physicochemical properties (Fig. 2h). Collectively, our analyses revealed that numerous peptides significantly more abundant in parental HEK293 CM stem from SPPL3-dependent intramembrane cleavage events, thus the parent type II membrane proteins can be confidently considered SPPL3 substrates (Fig. 2h). Presently, MGAT5 is among the few SPPL3 substrates with a known cleavage site (Met28|Leu29) [19, 20] and our data precisely confirm this scissile bond. In addition, we provide first cleavage site information for a number of previously reported substrates of SPPL3, including CANT1, B4GALT1, NDST1, POMK and B4GAT1, but we also confidently identify novel SPPL3 substrates, including GALNT2, FAM20B, CHST3 and others (Fig. 2h). Comparative analysis of peptides identified in HEK293 CM shows that substrate-derived N-terminal peptides differ in relative intensity, with numerous of the previously identified substrates being higher in abundance, potentially explaining why these have been dominant in previous datasets (Fig. 2i).

Notably, following our filtering, numerous N-terminal peptides were found to be significantly more abundant in CM of parental than of *SPPL3*-deficient HEK293 cells, yet did not reach our highly stringent fold-change cut-off (Fig. 2c, d). Given that the SPPL3-dependence of their secretion was significant, these peptides could still represent genuine SPPL3 cleavage products. This warranted closer inspection (Suppl. Figure 3c) and revealed that the majority of these peptides similarly mapped to type II membrane proteins predicted to localise to the Golgi and/or participate in cellular glycosylation pathways. Notably, we found unique peptides mapping to CHST14, GCNT1, B4GALNT1, MAN1C1 and XYLT2, which had been observed to be significantly altered in abundance following changes in SPPL3 expression in secretome data [20]. The peptides identified here confirm SPPL3-mediated intramembrane cleavage of these substrates (Suppl. Figure 3d). In addition, we found several examples of type II proteins (e.g. B3GAT3, XXYLT1 and others) represented by more than one TMD-derived N-terminal peptide, suggesting that these represent novel candidate SPPL3 substrates as well.

In an attempt to further improve detection of SPPL3 substrates, we isolated Golgi-derived vesicle-enriched subcellular fractions from *SPPL3*-deficient and parental HEK293 cells using discontinuous sucrose gradient centrifugation. In line with an expected Golgi localisation, endogenous SPPL3 co-segregated with established Golgi markers such as GS28 and GM130 in vesicles obtained from the 0.25/1.00 M sucrose interphase (Suppl. Figure 3e). As it was similarly enriched in GALNT2, a newly identified SPPL3 substrate (Fig. 2h), this fraction was subjected to mass spectrometric analyses to identify additional SPPL3 substrates. While N-terminome analysis was not insightful owing to the paucity of isolated material, label-free quantification of Golgi-enriched fractions revealed that select Golgi-resident type II membrane proteins accumulated in Golgi fractions from *SPPL3*-deficient cells (Suppl. Figure 3f). Apart from the previously reported substrates MGAT5 [19], B4GAT1 [19] and B3GNT5 [24, 25], the GTfs MGAT4A, B4GALT6 and B4GALT5 also accumulated in the Golgi fraction of *SPPL3*-deficient cells and thus represent additional substrate candidates.

### Identification of SPPL3 substrates in conditioned supernatants of isogenic knock-out HeLa cells

**Fig. 3:**
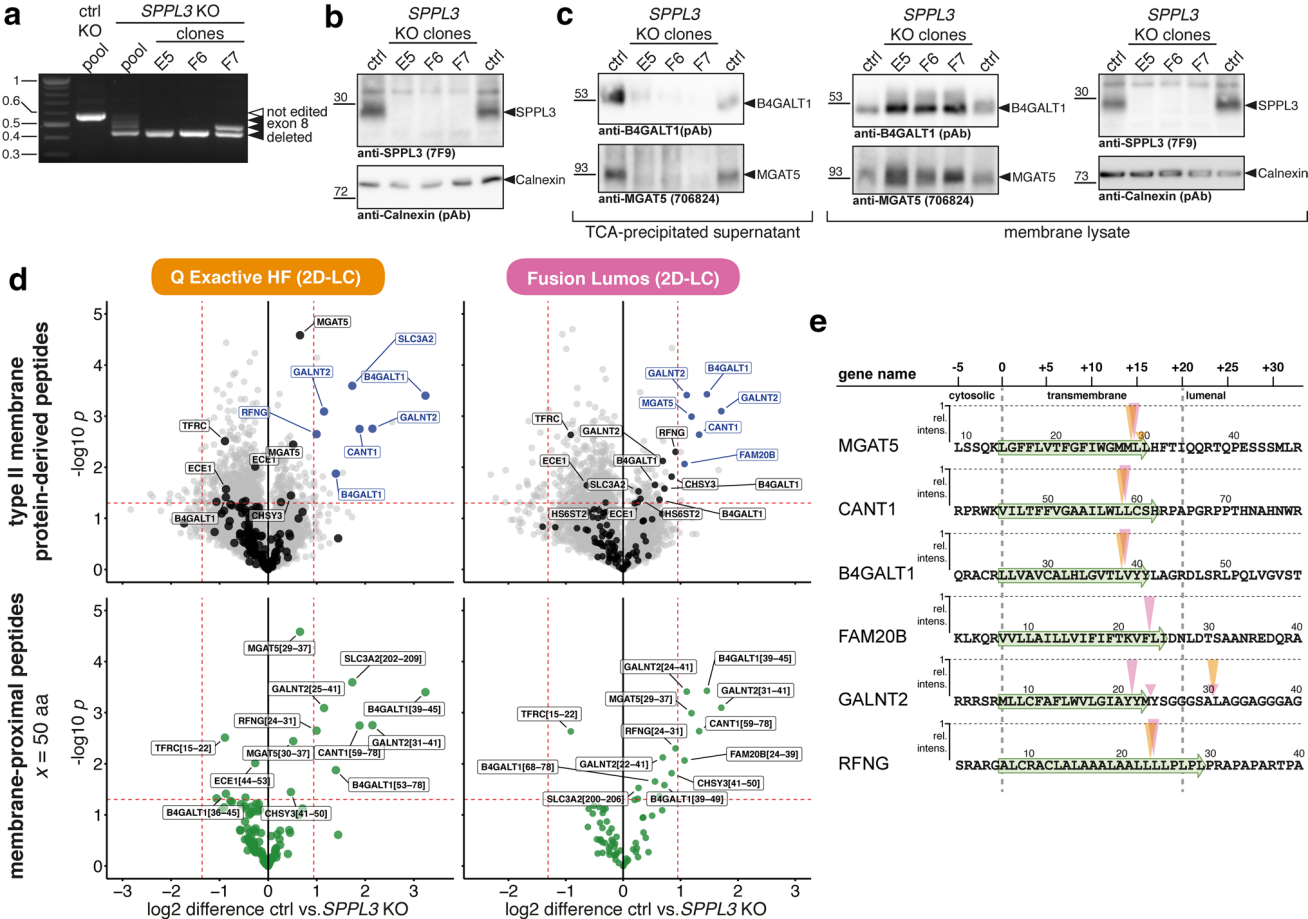
N-terminome analysis of CM of *SPPL3*-deficient isogenic HeLa cells. **a** PCR genotyping of the exon 8 region of the *SPPL3* locus in HeLa cells following Cas9 RNP electroporation. The parental 545 bp band was only detected in cells treated with sgRNAs directed against a control target locus. It was diminished in pooled cells following SPPL3 targeting and completely absent from *SPPL3* KO clones E5, F6, F7. Truncated PCR products demonstrate deletions within exon 8. **b** SPPL3 immunoblotting of carbonate-washed membranes obtained from *SPPL3* KO clones and parental HeLa cells (ctrl). **c** Detection of MGAT5 and B4GALT1 in CM and cellular membranes from *SPPL3* KO clones and parental HeLa cells. TCA-precipitated conditioned supernatants (left) and lysates of carbonate-washed cellular membranes (right) were subjected to immunoblotting with the indicated antibodies. Calnexin was used as a loading control for the membrane fraction. **d** Volcano plots showing peptides detected following HYTANE enrichment of CM from *SPPL3*-deficient clones and parental HeLa cells. Samples were analysed in parallel on the indicated LC–MS systems. 7864 and 5372 unique peptides are plotted, respectively and labelling/filtering is performed as outlined in Fig. 2e with *x* = 50 aa. Dashed lines represent threshold values (± 2 SD for log2 difference and *p* = 0.05). **e** Schematic overview and mapping of neo-N peptides found to be changed in abundance in CM (see Fig. 2h)

As evidenced by transcriptome data [36], *SPPL3* is ubiquitously expressed in most human cell lines, but Golgi glycosylation enzymes representing possible SPPL3 substrates may be differentially expressed. Hence, to confirm our findings in a different cellular setting and as transcriptome data point to a different expression pattern of genes encoding Golgi GTfs and other potential SPPL3 substrates (Suppl. Figure 4a), we generated isogenic SPPL3 KO HeLa cell clones following the same approach outlined in Fig. 1a. PCR amplification of the *SPPL3* exon 8 region from gDNA of successfully edited HeLa clones produced only truncated products with no traces of the 545 bp band corresponding to the native locus found in control cells electroporated with Cas9 RNPs targeting an unrelated locus (Fig. 3a). Sanger sequencing of PCR products again confirmed exon 8 deletions (Suppl. Figure 4b) and we did not detect mutations in predicted off-target sites (Suppl. Figure 5). SPPL3 could not be detected by immunoblotting of membrane lysates, while it was clearly expressed in parental HeLa cells (Fig. 3b). As for the HEK293 cell model, *SPPL3* KO in HeLa cells was associated with impaired secretion and concomitant intracellular accumulation of MGAT5 and B4GALT1 (Fig. 3c). We then conducted comparative N-terminome analyses of serum-free CM of the three isogenic HeLa *SPPL3* KO clones and parental cells (Fig. 3d, e). Also when analysing HeLa cell CM, we identified neo-N-terminal peptides that were more abundant in CM of parental cells and depleted from *SPPL3*-deficient cells, suggesting that proteoforms carrying these N-termini are secreted in a SPPL3-dependent fashion. Filtering revealed that these peptides again derived from TMD-proximal regions of type II membrane proteins and included the previously reported substrates MGAT5, B4GALT1 and CANT1 [19, 20], but also FAM20B, RFNG and GALNT2 identified as SPPL3 substrates in HEK293 cells (Fig. 2). For GALNT2, two peptides were significantly changed, the first ([24–41]) originating at the C-terminal tip of the annotated TMD and the second ([31–41]) in the adjacent luminal region, raising the possibility that the latter is generated following exopeptidase trimming of an initial SPPL3 cleavage product. Also in HEK293 cells, peptides mapping to several distinct potential cleavage sites in GALNT2 were observed (Fig. 2h). Importantly, with the exception of GALNT2, cleavage sites identified for substrates of endogenous SPPL3 in HeLa cells are identical to those identified in HEK293 cells (e.g. MGAT5 (Met28|Leu29), B4GALT1 (Leu38|Val39), CANT1 (Leu58|Leu59) and FAM20B (Phe23|Leu24); Figs. 2h, 3e).

### N-terminome analysis of HEK293 cells ectopically expressing SPPL3

**Fig. 4:**
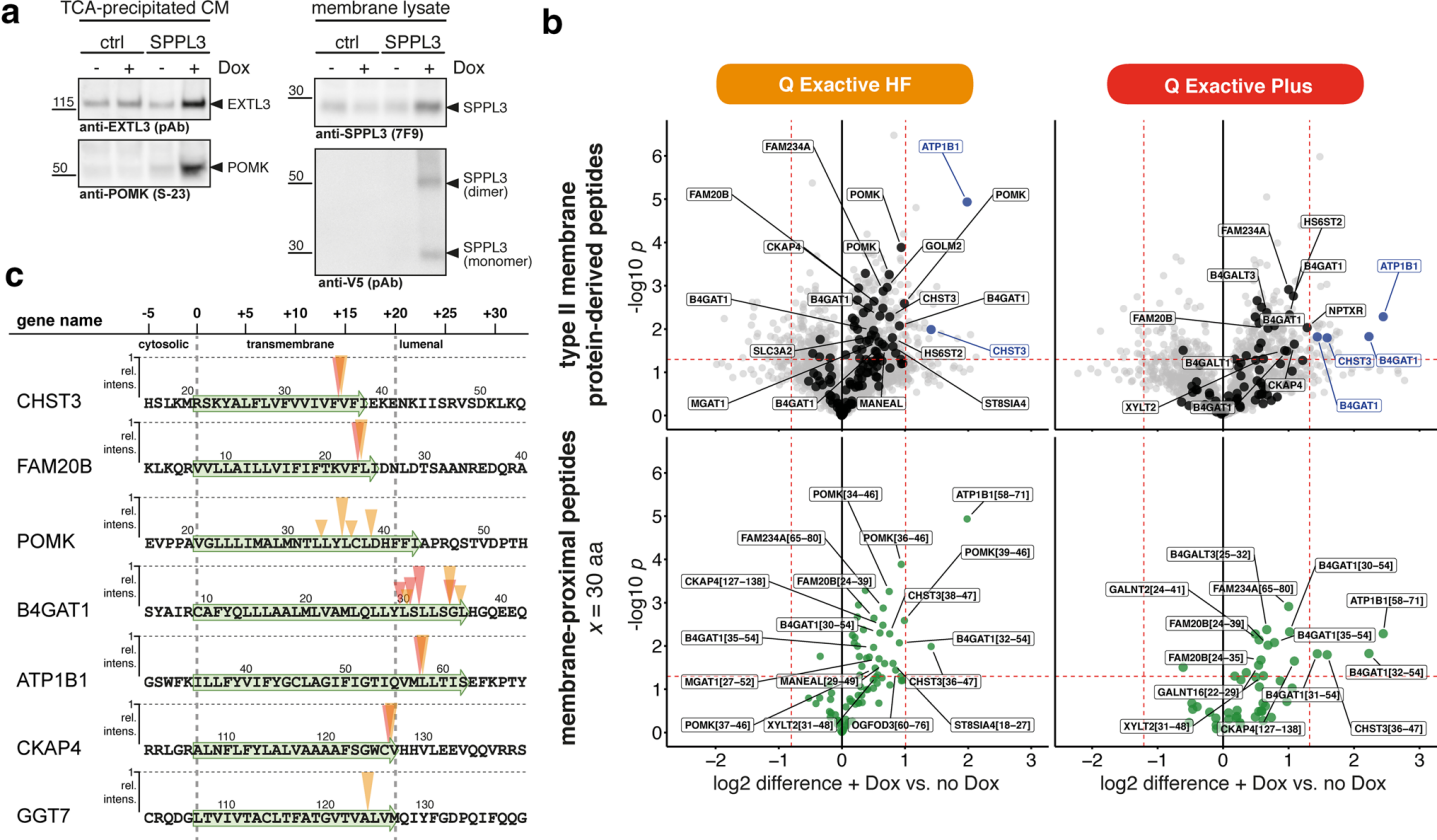
N-terminome analysis of CM of HEK293 cells overexpressing SPPL3. **a** Immunoblots of TCA-precipitated CM (left) and lysates of carbonate-washed membranes (right) of untransfected (ctrl) and SPPL3-V5 WT-transfected Flp-In™ T-REx™ HEK293 cells. Treatment with doxycycline (Dox) induces SPPL3 overexpression and leads to increased release of EXTL3 and POMK into the CM. **b** Volcano plots showing peptides detected following HYTANE enrichment of CM from doxycycline-treated and untreated Flp-In™ T-REx™ SPPL3-V5 HEK293 cells (three technical replicates). Samples were analysed in parallel on the indicated LC–MS systems. 2,817 and 1,608 unique N-terminal peptides are plotted, respectively, and labelling/filtering is performed as outlined in Fig. 2e with *x* = 30 aa. Dashed lines represent threshold values (± 2 SD for log2 difference and *p* = 0.05). **c** Schematic overview and mapping of neo-N peptides found to be changed in abundance in CM (see Fig. 2h). Arrow height indicates relative intensities (mean of doxycycline-treated samples) for membrane-proximal peptides changed upon SPPL3 overexpression

Our terminome analyses in isogenic *SPPL3*-deficient cells did not verify all previously reported SPPL3 substrates, possibly since these were initially uncovered using more sensitive approaches such as candidate-based immunoblotting [19], enrichment through metabolic labelling [20] or high-throughput genetic screening [24]. To capture additional neo-N-terminal peptides generated by SPPL3 activity, we thus turned to a SPPL3 overexpression system, which is expected to boost substrate release into the CM (yet might have limited physiological significance). We used stably transfected Flp-In™ T-REx™ HEK293 cells with C-terminally V5-tagged SPPL3. Through doxycycline treatment a mild SPPL3 overexpression can be achieved as demonstrated by immunoblotting and is accompanied by increased secretion of the GTf EXTL3 and the kinase POMK (Fig. 4a), as observed earlier [20]. We then performed comparative analysis of N-terminal peptides enriched via HYTANE from CM of Flp-In™ T-REx™ SPPL3-V5 WT cells treated with doxycycline (i.e. overexpressing SPPL3) and untreated cells (no SPPL3 overexpression) (Fig. 4b). Applying the same filtering outlined earlier with a high stringency for membrane-proximity, we observed that overexpression of active SPPL3 led to a globally increased release of type II membrane protein-derived TMD-proximal peptides, though only three peptides surpassed the fold-change cut-off set. These included peptides derived from the described substrates B4GAT1 [19], CHST3 (this study, Figs. 2, 3) and ATP1B1. While the analysis could not reveal novel high-confidence substrates of overexpressed SPPL3 with a fold-change > 2 SD, several N-terminal peptides from previously identified SPPL3 substrates were significantly right-shifted, indicating SPPL3-dependent release. We could thus confirm the cleavage sites for CHST3 and FAM20B (Figs. 2, 3). ATP1B1, a regulatory subunit of the cell surface Na^+^/K^+^ ATPase, was previously found to be more abundant in CM upon SPPL3 overexpression and an incidentally identified semi-tryptic peptide suggested SPPL3-mediated cleavage at Met57|Leu58 [20]. A corresponding peptide was identified here, confirming this cleavage site (Fig. 4c). In addition, cleavage sites of CKAP4 and GGT7, two additional substrates of overexpressed SPPL3 reported before [20], could be identified at Cys126|Val127 and Ala124|Leu125, respectively. For POMK and B4GAT1 multiple peptides with an origin in the TMD became particularly prominent following SPPL3 overexpression with select peptides corresponding to the POMK and B4GAT1 cleavage sites identified for endogenous SPPL3 (Fig. 2h).

### Processing of novel SPPL3 substrates is dependent on endogenous SPPL3 protease activity

**Fig. 5:**
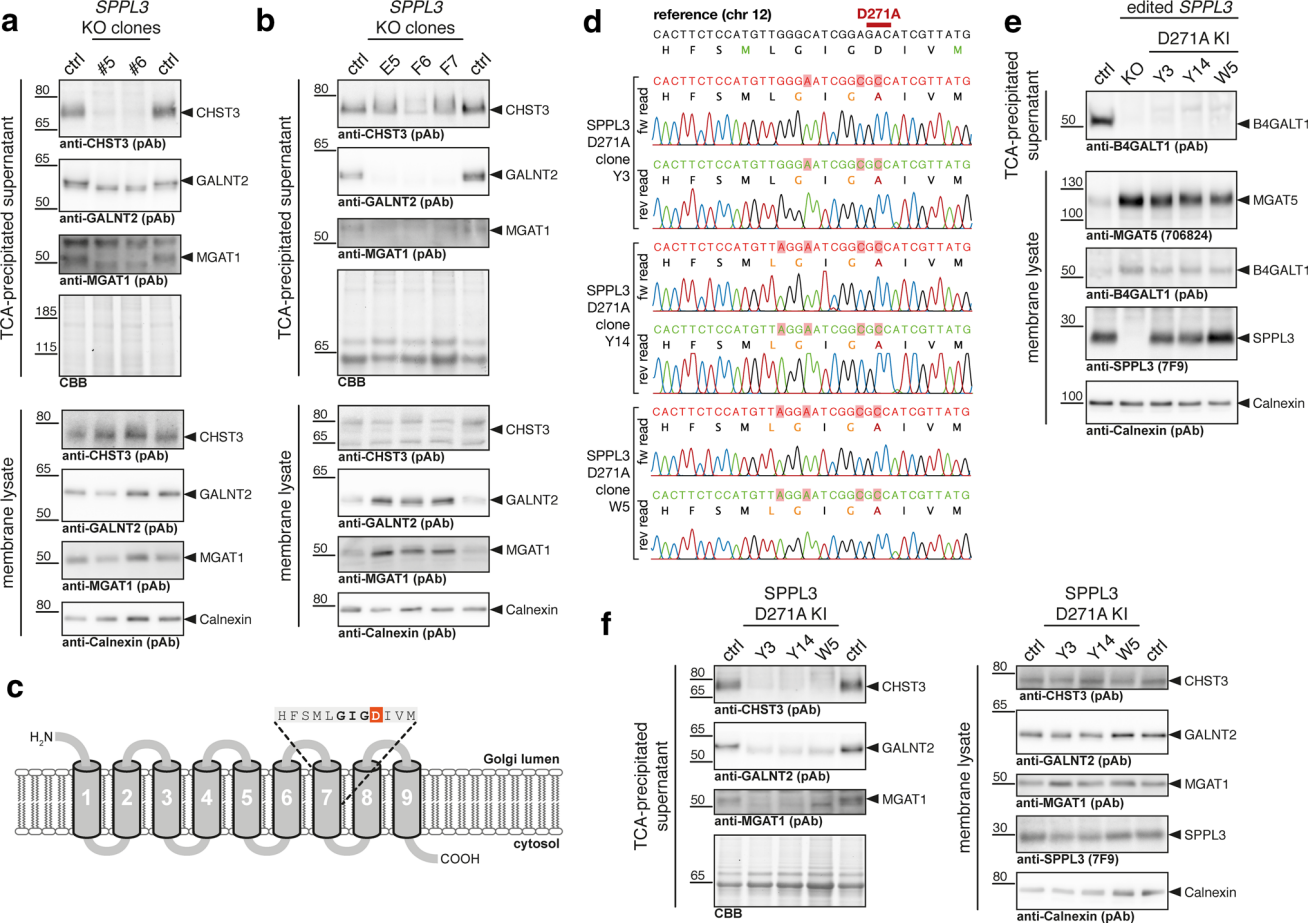
Validation of novel SPPL3 substrates in *SPPL3* KO and SPPL3 D271A knock-in cells. **a** Immunoblot validation of the newly identified SPPL3 substrates CHST3, GALNT2 and MGAT1 in HEK293 cells. TCA-precipitated CM and membrane lysates obtained from three distinct *SPPL3* KO clones and parental HEK293 cells (ctrl) were probed using specific antibodies. Calnexin served as loading control for the membrane fraction, total protein staining with Coomassie Brilliant Blue (CBB) as loading control for CM samples. **b** Immunoblot validation of the CHST3, GALNT2 and MGAT1 in HeLa cells. **c** Overview of SPPL3 topology (loops are not to scale). D271 targeted is highlighted in red, the GIGD motif in bold font. **d** Sanger sequencing reads confirming correct genome editing in *SPPL3* D271A knock-in HEK293 cells. The targeted region of the *SPPL3* locus was amplified by PCR from genomic DNA isolated from the indicated clones, gel-purified and Sanger sequenced. Reads were aligned to the corresponding chromosome 12 reference sequence. Encoded SPPL3 primary structure is provided for the correct reading frame. Mutated residues are highlighted by a red background. All clones analysed carry only the desired D271A missense mutation (red aa residue) in the active site of SPPL3. Additional mutations were introduced to prevent Cas9 re-cleavage, but remained silent (orange aa residues). **e** Phenotypic validation of SPPL3 D271A knock-in cells in comparison to *SPPL3* KO and parental HEK293 cells. Secretion of B4GALT1 from cells was examined by immunoblotting of TCA-precipitated samples. Lysates of carbonate-washed membranes from the indicated cell clones were probed for the SPPL3 substrate MGAT5 and B4GALT1 as well as for SPPL3. **f** Immunoblot analysis of CHST3, GALNT2 and MGAT1 levels in TCA-precipitated supernatant and membrane lysates of isogenic SPPL3 D271A HEK293 cells

Our analyses in HEK293 and HeLa cells identified numerous proteins that are (1) type II membrane proteins, (2) secreted in a manner dependent on endogenous SPPL3 and (3) generated through endoproteolysis within their annotated TMD. These can thus be confidently considered SPPL3 substrates. To further corroborate our proteome data, we sought to validate novel SPPL3 substrates using immunoblotting, yet were only able to obtain specific antibodies that displayed sufficient sensitivity to detect endogenous levels of CHST3, GALNT2 and MGAT1. We assessed levels of the secreted GTf proteoforms in TCA-precipitated CM of *SPPL3*-deficient isogenic HEK293 cells and parental control cells and in parallel analysed levels of the unprocessed membrane-anchored GTf proteoforms in membrane lysates (Fig. 5a). In line with our N-terminome data, immunoblotting confirmed that secretion of CHST3, GALNT2 and MGAT1 from *SPPL3*-deficient HEK293 cells was reduced significantly compared to parental cells (Fig. 5a and Suppl. Figure 6a). This demonstrates that CHST3, GALNT2 and MGAT1 are subject to continuous SPPL3-dependent secretion in HEK293 cells. Contrasting with previous observations for MGAT5 and B4GALT1 (Fig. 1d), we did, however, not observe a marked accumulation of the newly identified substrates in the membrane fraction of SPPL3-deficient HEK293 cells. We also assessed secretion of two additional GMEs, B3GAT3 and CHST14, but due to a lack of specific antibodies detecting endogenous proteins levels, we transfected HEK293 cells with C-terminally V5-tagged B3GAT3 and CHST14, respectively, to monitor their secretion. Overexpressed B3GAT3 was secreted from HEK293 transfectants, but secretion was reduced in *SPPL3*-deficient cells and unprocessed B3GAT3 was even found to accumulate intracellularly (Suppl. Figure 6b). CHST14 was previously reported as a candidate substrate [20] and, supporting this, we observed a TMD-derived neo-N-terminal peptide that was generated in a SPPL3-dependent fashion (Suppl. Figure 3c, d). Mirroring our observations for B3GAT3, loss of SPPL3 in HEK293 cells transfected with V5-tagged CHST14 led to marked reduction of CM CHST14 and was also associated with intracellular CHST14 accumulation, but secretion was not completely abolished (Suppl. Figure 6c), suggesting (an)other protease(s) can also facilitate release of B3GAT3 and CHST14, either constitutively or as part of a compensatory mechanism in *SPPL3*-deficient cells. Interestingly, we detected another, SPPL3-independent neo-N-terminal CHST14 peptide in our terminome dataset that originates in the luminal stem region and could be the result of such alternative cleavage (Suppl. Figure 6d). We similarly assessed CHST3, GALNT2 and MGAT1 secretion in *SPPL3*-deficient HeLa cells. Here, a robust loss of GALNT2 secretion was noted (Fig. 5b and Suppl. Figure 7a), which was also accompanied by an intracellular accumulation of GALNT2 (Fig. 5b), strongly supporting that SPPL3 is critically required to facilitate secretion of GALNT2 in HeLa cells. This could also be confirmed using a different GALNT2-specific antibody (Suppl. Figure 7b). A similar effect of the SPPL3 KO in HeLa cells was noted for MGAT1 (Fig. 5b and Suppl. Figure 7a). Contrasting with the situation in HEK293 cells, HeLa cell CHST3 secretion was not significantly reduced by loss of SPPL3 (though, as a heavily glycosylated protein, CHST3 displayed a markedly changed running behaviour due to prevalent hyperglycosylation in cells lacking SPPL3 [19]). While this suggests that SPPL3 universally impacts Golgi enzyme secretion, the contribution of SPPL3 and other proteases to this process may differ in a cell type-specific manner.

Of note, SPPL3 was also reported to impact calcium signalling in T cells independent of its proteolytic activity [37]. As SPPL3 expression is completely abrogated in KO cell lines, these cannot aid in differentiating between proteolytic and non-proteolytic effects of SPPL3. Therefore, to further strengthen that Golgi enzyme cleavage is strictly dependent on proteolytic activity of endogenous SPPL3, we targeted the catalytic GIGD motif in TMD7 of SPPL3 through precise genome editing and generated knock-in HEK293 cells expressing the active site SPPL3 mutant D271A (Fig. 5c). This mutant was previously reported to display impaired proteolytic activity upon overexpression [21]. Following electroporation of HEK293 cells with *SPPL3* exon 9-directed Cas9 RNPs and a 90-mer ssODN repair template, we established three HEK293 clones that were homozygous for the desired SPPL3 D271A mutant as demonstrated by Sanger sequencing (Fig. 5d). As expected, SPPL3 was expressed in knock-in clones but its activity was clearly impaired because unprocessed MGAT5 and B4GALT1 accumulated in the membrane fraction and B4GALT1 was depleted from CM similar to *SPPL3* KO cells (Fig. 5e). Finally, we also probed TCA-precipitated CM collected and membranes isolated from the three SPPL3 D271A HEK293 clones for the newly identified substrates CHST3, GALNT2 and MGAT1 (Fig. 5f and Suppl. Figure 7c). Recapitulating our observations in isogenic SPPL3 KO HEK293 cells (Fig. 5a), we found that secretion of CHST3, GALNT2 and MGAT1 was impaired in all three clones expressing exclusively SPPL3 D271A (Fig. 5f and Suppl. Figure 7c), demonstrating that catalytic activity of SPPL3 is strictly required and further supporting that intramembrane proteolysis catalysed by SPPL3 is the rate-limiting step in B4GALT1, CHST3, GALNT2 and MGAT1 secretion.

### Substrate TMDs can contain critical determinants for SPPL3 endoproteolysis

**Fig. 6:**
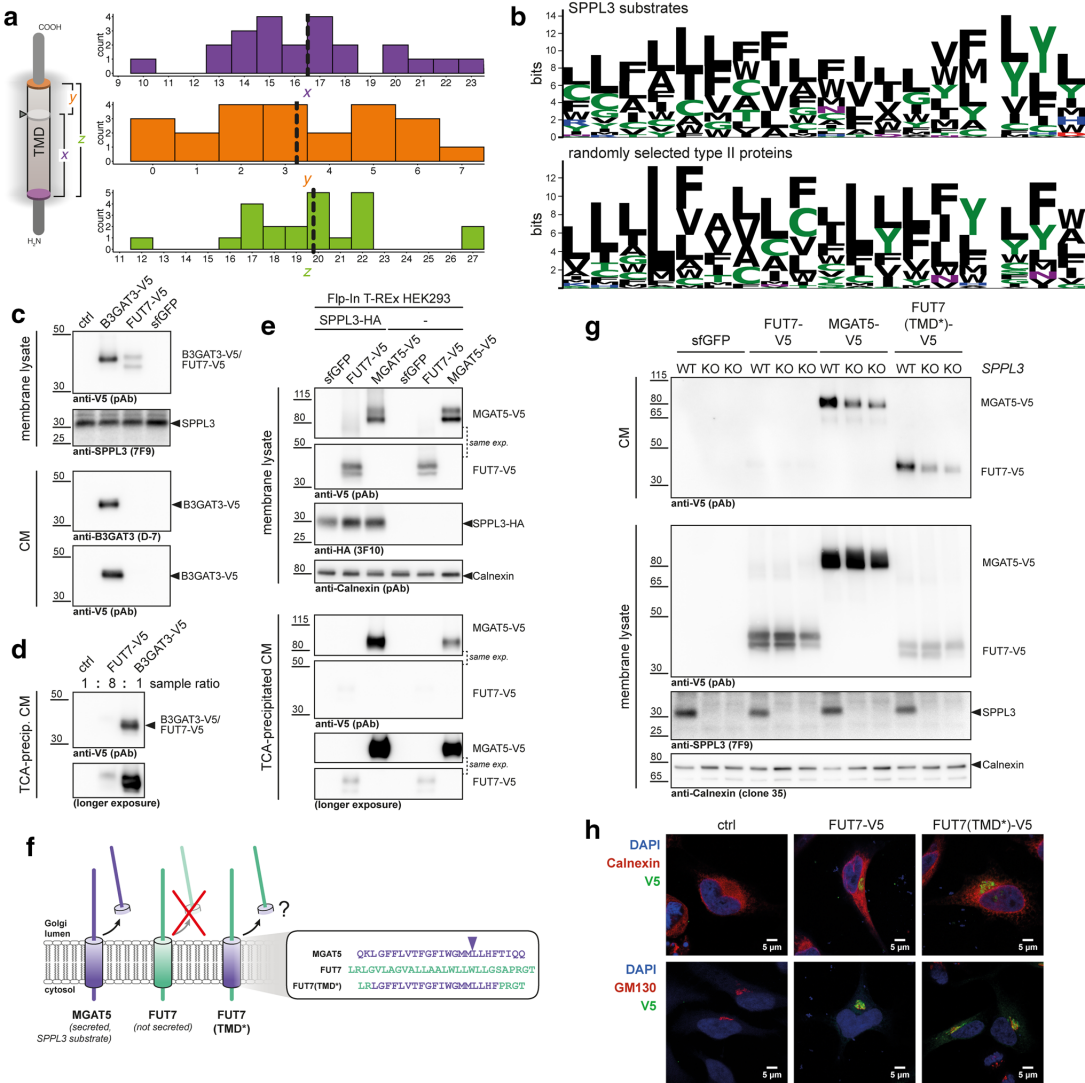
Substrate TMDs are critical determinants of SPPL3-mediated substrate endoproteolysis. **a** Cleavage site position in relation to the TMD of the substrate. (left) Schematic overview of analysis performed. Distance (in aa) from a SPPL3 cleavage site (grey disc and arrowhead) to the first residue of the TMD (purple disc, *x*; as annotated in Uniprot) and to the last annotated residue of the TMD (orange disc, *y*) as well as the total TMD length (green, *z*) were calculated. (right) Histogram plots showing the calculated values for 23 confidently identified SPPL3 cleavage sites (Suppl. Tables). Dashed lines represent mean values. **b** Sequence logo plots visualising PSSMs calculated for SPPL3 substrates with a confidently identified cleavage site (top panel; *n* = 23, Suppl. Tables) and 23 randomly selected type II Golgi proteins (bottom panel). For SPPL3 substrates, input sequences comprise P15 to P3’, i.e. are aligned based on the experimentally determined cleavage site (gap). In the bottom panel, taking *y* ≈ 3 (**a**) into consideration, peptides comprise the last annotated aa of the TMD as well as the preceding 17 aa. **c** Immunoblots demonstrating that FUT7-V5 is not released into CM from HEK293 transfectants. B3GAT3-V5 is secreted and served as a control. **d** CM samples from **c** were enriched via TCA precipitation. An eightfold excess of CM of FUT7-V5 transfected cells was loaded. **e** FUT7-V5 is not released from cells following SPPL3 overexpression. Flp-In T-Rex HEK293 cells and Flp-In T-Rex HEK293 stably transfected with SPPL3-HA were treated with doxycycline and transfected transiently as indicated. Secretion of GTfs was monitored in the CM. The known SPPL3 substrate MGAT5-V5 served as control. same exp., panels cropped from one identical image acquired. **f** Schematic overview of the FUT7 chimeric construct, FUT7(TMD*), generated. MGAT5 is known to be proteolytically cleaved and secreted; FUT7 is neither cleaved nor secreted. Arrowhead indicates the experimentally determined SPPL3 cleavage site in MGAT5. Note that all constructs contained a C-terminal V5 tag for simultaneous detection. **g** FUT7(TMD*) is secreted from transiently transfected HeLa cells in a SPPL3-dependent fashion. Parental HeLa cells (WT) as well as *SPPL3*-deficient HeLa clones F6 and F7 were transfected as indicated and expression and release of GTf constructs was monitored by anti-V5 immunoblotting. **h** Confocal microscopy of HeLa cells transfected with FUT7-V5 and FUT7(TMD*)-V5 and stained with an anti-V5 mAb as well as antibodies against the ER and Golgi markers Calnexin and GM130, respectively. Non-transfected (ctrl) cells displayed no anti-V5 staining

Notably, our study provides the first comprehensive list of SPPL3 cleavage sites and thus enables novel mechanistical insights into SPPL3-mediated type II membrane protein cleavage. We first examined where scissile bonds within SPPL3 substrates are located in relation to the predicted/annotated position of their TMD (Fig. 6a). Based on Uniprot annotations, TMDs of SPPL3 substrates had an average length of 20 aa (outliers: ST8SIA4 with very short and ATP1B1 and B4GAT1 with very long annotated TMDs). Cleavage occurred on average 16–17 aa after the first residue of the TMD and within the exoplasmic half, but not outside of the TMDs. To identify motifs potentially enabling SPPL3 cleavage, we, therefore, asked whether the TMDs of SPPL3 substrates share certain sequence features. To this end, we retrieved substrate protein sequences spanning position P15 to P3’ (i.e. comprising the bulk of the TMD) and calculated a position-specific scoring matrix (PSSM). As evident from its visualisation in Fig. 6b and in general agreement with expectations for TMDs [38], hydrophobic aa residues were predominant in SPPL3 substrates, in particular in proximity to the scissile bonds. In this region an enrichment of Tyr residues was also noted. Both features, however, became also apparent when we similarly analysed a corresponding region of randomly selected Golgi type II membrane proteins (which excluded SPPL3 substrates). To more closely look for shared sequence motifs in the vicinity of identified SPPL3 cleavage sites we also used the IceLogo algorithm [39] and scored our substrate peptides containing scissile bonds (P15 to P3’) against all or only Golgi-localized type II membrane protein TMDs (Suppl. Figure 8a, b). The analyses revealed only a modest enrichment of aa residues in SPPL3 substrates compared to the reference datasets, most notably the presence of Gly residues in the centre and of Trp residues in the cytoplasm-facing region of the TMDs. Regarding the former, it needs to be noted that TMD Gly residues were found to be relevant for intramembrane proteolysis catalysed by the related intramembrane proteases SPPL2 and SPPL2b [40, 41]. However, the relative abundance of these seemingly enriched residues was low among SPPL3 substrate peptides, i.e. they are only present in a minor fraction of SPPL3 substrates and thus unlikely to define a consensus cleavage or recognition motif. To further extend our analysis and overcome issues of ambiguous cleavage sites observed within SPPL3 substrates (Fig. 2h), we scored the last 18 aa of the TMDs of confidently identified SPPL3 substrates against the corresponding peptides of all human type II membrane proteins (Suppl. Figure 8c), but also this analysis did not reveal any obvious differences. We hence concluded that our cleavage site dataset does not reveal any overt patterns in primary structure required for SPPL3 endoproteolysis beyond the properties generally displayed by TMDs [38], raising the possibility that rather structural properties of substrate TMDs or other substrate features dictate cleavage by SPPL3.

To examine experimentally whether properties intrinsic to TMD sequences are relevant to SPPL3-mediated endoproteolysis, we turned our attention to the fucosyltransferase FUT7. Like most other Golgi GTfs, FUT7 is a type II membrane protein, but—unlike other GTfs—FUT7 is not cleaved and secreted [42]. It can, however, be turned into a secreted GTf once a large portion of FUT7 comprising the cytosolic N-terminus, its TMD and the luminal stem region are replaced by the corresponding region of FUT6 or ST6GAL1 [42, 43]. As HEK293 and HeLa cells do not express endogenous FUT7 [36], we cloned and overexpressed C-terminally V5-tagged FUT7 in HEK293 cells. While another tagged GTf, the SPPL3 substrate B3GAT3-V5 (Suppl. Figure 6b), was readily secreted into CM of transfectants, we were unable to detect secreted FUT7-V5 (Fig. 6c). Even following enrichment of secreted proteins by TCA precipitation we did not observe substantial secretion of FUT7-V5 and only when loading an eightfold excess we captured a faint FUT7-V5 signal which may be attributed to cellular debris or the like (Fig. 6d). When stably overexpressed in HEK293 cells, SPPL3 led to an increase in MGAT5-V5 secretion, but FUT7-V5 was not secreted in comparable amounts and faint FUT7-V5 signals in TCA-precipitated CM were not affected by SPPL3 overexpression (Fig. 6e), confirming that FUT7 is neither a SPPL3 substrate (not even when SPPL3 is overexpressed) nor cleaved by any other protease that enables subsequent secretion under our experimental conditions. To test whether the TMD of a SPPL3 substrate can harbour cleavage-determining properties we generated a C-terminally V5-tagged chimeric FUT7 construct, FUT7(TMD*), in which we replaced the intrinsic FUT7 TMD with the TMD of the SPPL3 substrate MGAT5 (Fig. 6f) using a seamless cloning strategy. Along with the parental FUT7 and MGAT5 constructs, FUT7(TMD*) was then transiently transfected into HeLa cells (Fig. 6g). As in HEK293 cells (Fig. 6c, d), FUT7 was detected in lysates of cellular membranes but was not released into CM in substantial amounts. In striking contrast to this, yet similar to MGAT5, FUT7(TMD*) was present in CM suggesting it had been proteolytically cleaved and secreted. As expected [19], MGAT5 secretion was reduced in *SPPL3* KO HeLa cells, but it was not completely abrogated suggesting (an)other protease(s) also contribute(s) to its cleavage or can compensate for the lack of SPPL3. Notably, secretion of FUT7(TMD*) displayed a SPPL3-dependency similar to MGAT5. To exclude that these strikingly different properties of FUT7 and FUT7(TMD*) are merely related to differences in subcellular localisation, we performed confocal microscopy and found that both constructs displayed identical subcellular localisation, similarly localising to a distinctive juxtanuclear region stained by the Golgi marker GM130 and not displaying overt co-localisation with the ER marker Calnexin (Fig. 6h). Our results thus demonstrate that features intrinsic to the TMD of MGAT5 alone can enable SPPL3-dependent secretion of a protein not naturally cleaved by SPPL3 (and even resistant to SPPL3 overexpression).

### SPPL3 cleavage products are present in human blood

**Fig. 7:**
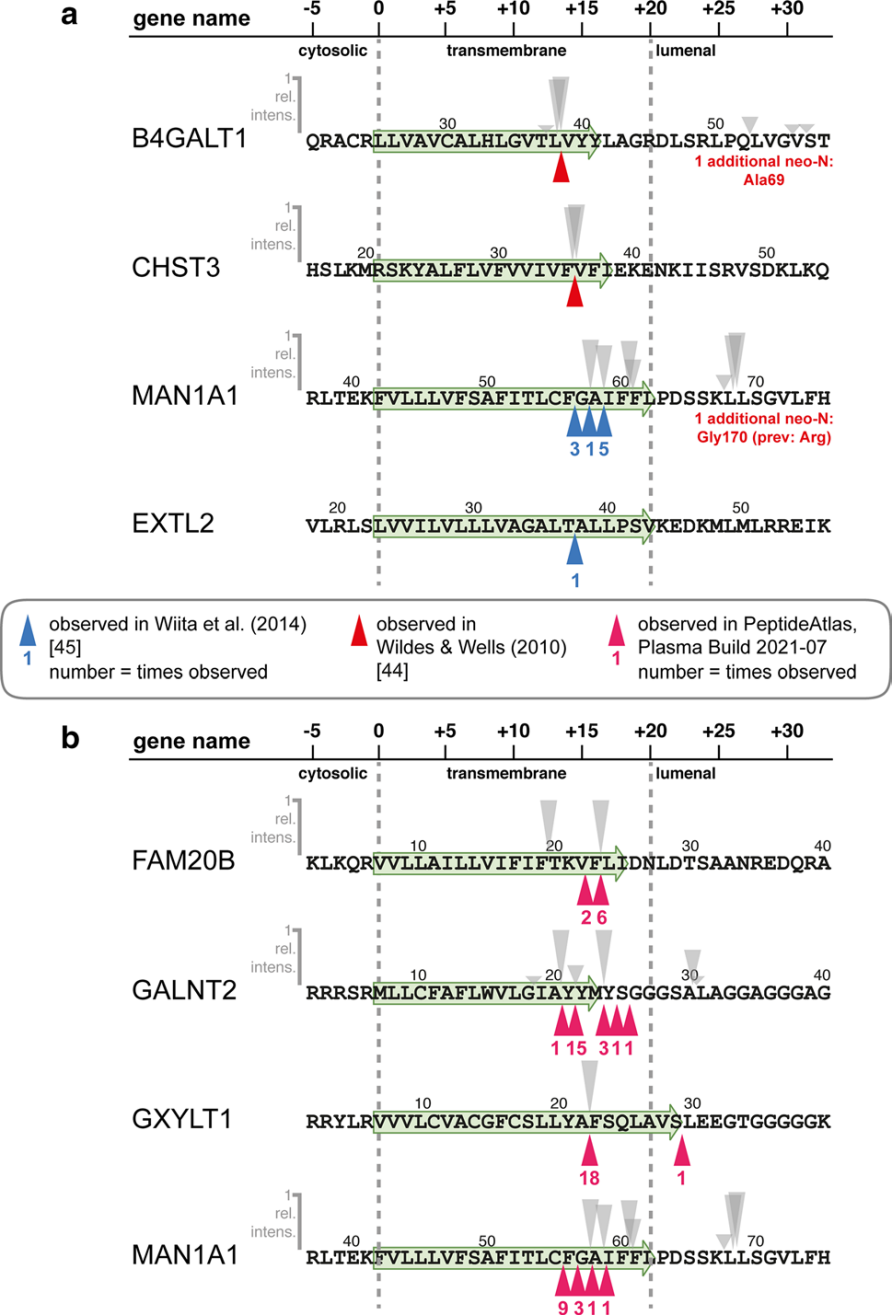
SPPL3 cleavage products detected in human blood proteome data. **a** Intramembrane cleavage sites inferred from neo-N-termini identified in degradome set of human serum. Cleavage sites mapped to select type II membrane protein Golgi GMEs are depicted. Grey arrows indicate SPPL3-depedent proteolytic events identified in this study (Fig. 2), red and blue arrowheads indicate cleavage sites observed in blood samples. **b** Intramembrane cleavage sites inferred from semi-tryptic peptides deposited in the Human Plasma PeptideAtlas (Build 2021-07). The deposited data were searched for Golgi GMEs that we found to undergo SPPL3-mediated intramembrane proteolysis in this study. Peptide accession numbers are compiled in the Suppl. Tables

We identified numerous neo-N-terminal peptides that were secreted from cells in a SPPL3-dependent fashion in vitro. Neo-N-termini identified in HEK293 and HeLa cells were mostly identical suggesting universal use of these cleavage sites by endogenous SPPL3. Considering their unique and distinctive properties—originating exclusively from cleavage within the lumen-facing third of the TMD of a (Golgi-localised) type II membrane protein—these neo-N-termini represent “signatures” of SPPL3-mediated intramembrane proteolysis and would allow specific and confident identification of SPPL3 cleavage products. Golgi GTfs are known to be present in body fluids such as blood [5, 6] and we consequently surveyed public data for SPPL3 cleavage signatures. First, we inspected human serum degradome data obtained following subtiligase labelling and positive enrichment of neo-N-termini [44, 45] and filtered for GMEs with a type II topology or established SPPL3 substrates (Suppl. Tables). Even though the datasets only contained cleavage site information for a few of these proteins (B4GALT1, CHST3, EXTL2, GXYLT1, HS3ST3B1 & MAN1A1), the generation of their detected cleavage products in vivo can be mainly attributed to intramembrane proteolysis (Fig. 7a). In fact, for B4GALT1 and CHST3 neo-N-termini matching precisely with the cleavage products we identified in HEK293 (Fig. 2h) [and for B4GALT1 also in HeLa (Fig. 3e)] cells were reported. Since we found in our cell culture models that secretion of these N-termini was evidently dependent on endogenous SPPL3, this demonstrates that SPPL3 cleavage products of B4GALT1 and CHST3 can be found in vivo in human blood. This similarly applies to MAN1A1, for which three distinct, yet consecutive TMD-derived N-termini had been found, matching in parts with the SPPL3-dependent peptides we observed. For EXTL2, a prominent GME in blood [6], an identified neo-N-terminus similarly suggests that EXTL2 is released by an intramembrane protease, thus it could also constitute a SPPL3 substrate. To further corroborate the SPPL3-dependent secretion of Golgi enzymes into human blood in vivo and to overcome limitations caused by the confined depth of the blood degradome data, we next surveyed the most recent build of the *Human Plasma PeptideAtlas* (Build 2021-07) for semi-tryptic peptides observed specifically for Golgi-resident type II membrane proteins and deriving from membrane-spanning regions. Indeed, for FAM20B, GALNT2, GXYLT1 and MAN1A1, which we identified as novel SPPL3 substrates in this study, deposited data included semi-tryptic peptides originating within the substrates’ TMDs (Fig. 7b) suggesting that SPPL3 facilitates secretion of these enzymes into human blood. All these peptides commence within the second half of the TMD and in all cases also include peptides that we identified following negative enrichment of neo-N-terminal peptides from HEK293 and HeLa cell CM, though additional peptides were also identified in blood. For GXYLT1, for instance, a robustly identified semi-tryptic peptide in the database (18/270 peptide observations) corresponds precisely to an N-terminus that we found to be generated in a SPPL3-dependent fashion in HEK293 cells (Suppl. Figure 3c). Collectively, this suggests that SPPL3-mediated intramembrane proteolysis contributes to the pool of secreted B4GALT1, CHST3, FAM20B, GALNT2, GXYLT1 and MAN1A1 present in human plasma in vivo. In addition, the *Human Plasma PeptideAtlas* contained several additional semi-tryptic peptides for Golgi enzymes (including B3GNT2, B3GNT8, ST3GAL6 and others) that may similarly represent cleavage products of intramembrane proteolysis, potentially even of SPPL3 (Suppl. Figure 9a). Additional peptides mapping in close proximity of the TMD (within 10 aa following the last annotated TMD residue) of Golgi enzymes were similarly identified (Suppl. Figure 9b). Semi-tryptic peptides mapping to this region could be generated by exopeptidase trimming after SPPL3 intramembrane cleavage and membrane-proximal tryptic peptides provide indirect evidence of intramembrane cleavage. In sum, these peptide data further corroborate that intramembrane (likely catalysed chiefly by SPPL3) or membrane-proximal endoproteolysis is a major source of secreted Golgi enzymes found in human blood and possibly the extracellular space in general.

## Discussion

### SPPL3 substrate spectrum

Pursuing an N-terminomic approach, our study, along with others [20, 22–25, 34], corroborates the concept that SPPL3 facilitates secretion of select GTfs and other GMEs in the Golgi network [19]. Previously, in a first proteomic approach to identify SPPL3 substrates, Kuhn et al. exploited metabolic glycan labelling to enrich de novo synthesized glycoproteins from serum-containing CM and found that secretion of several type II proteins was SPPL3-dependent [20]. Such substrate identification based on metabolic glycan labelling may, however, be complicated by the fact that SPPL3 alters cellular glycosylation in a complex and not completely understood manner [22, 24, 34]. Moreover, apart from few detected semi-tryptic peptides, the previous study did not provide insights in respect to SPPL3 cleavage sites. We now report on multiple proteomic datasets that add novel proteins to the list of identified substrates of endogenous SPPL3. Notably, our study identifies numerous neo-N-termini secreted in a SPPL3-dependent fashion. The fact that these derive from endoproteolysis within annotated TMDs strongly substantiates direct intramembrane cleavage catalysed by SPPL3. Suppl. Figure 10 provides an overview of proteins we consider genuine SPPL3 substrates and summarises the evidence supporting this.

While the majority of substrates identified for endogenous SPPL3 are Golgi-resident enzymes implicated in Golgi glycosylation pathways, our N-terminome analysis of SPPL3 overexpressing cells revealed additional other, non-Golgi type II proteins as SPPL3 substrates (e.g. ATP1B1, CKAP4 and GGT7, which were also identified by Kuhn et al. [20]). As we find no evidence of intramembrane cleavage of these proteins in cells with endogenous SPPL3, we assume that overexpressed SPPL3 is less strictly compartmentalized than endogenous SPPL3 and thus can access non-Golgi protein substrates with matching substrate properties or cleave proteins during Golgi transit. Interestingly, CKAP4 appears to have a dual biological function: the majority of CKAP4 is ER-resident and mediates ER-microtubule interactions but CKAP4 is also present at the plasma membrane and acts as receptor for Dickkopf1 (Dkk1), a secreted antagonist of Wnt signalling [46]. The Dkk1-CKAP4 signalling axis can drive cell proliferation and Dkk1 as well as CKAP4 are upregulated in tumours. Through cleavage of CKAP4, SPPL3 could theoretically antagonize CKAP4 signalling.

Despite our identification of novel substrates, we assume that the list of physiological SPPL3 substrates is not complete, in particular because our analysis of plasma proteome data suggests that other Golgi enzymes are secreted following intramembrane proteolysis as well. In addition, one caveat of both the previous [20] and our study is that they are limited to the analysis of CM, i.e. neglect cleavage products that either are rapidly degraded once secreted or that are not secreted following cleavage. It is conceivable that, once cleaved off their TMD in the Golgi, select SPPL3 substrates are cleared via the endolysosomal system. With the rationale that it would capture cleaved SPPL3 substrates before they are cleared from the cell via secretion or lysosomal degradation, our proteomic analysis of Golgi-enriched sub-cellular fractions aimed to overcome this limitation, yet insufficient material was obtained for in-depth analysis.

### SPPL3 cleavage sites and substrate properties determining SPPL3 cleavage

Prior to this study knowledge of SPPL3 cleavage sites was very limited and based on peptide mass fingerprinting of overexpressed, truncated substrates [19] or incidentally identified semi-tryptic peptides [20]. Our present study provides a comprehensive account of more than 20 cleavage sites, allowing first insights into the molecular mechanisms underlying intramembrane proteolysis catalysed by SPPL3. Demonstrating true intramembrane cleavage, we found that SPPL3 cleavage occurs within the lumen-facing third of the TMD. Cleavage site information at hand, we examined whether there is evidence of a potential consensus cleavage site of SPPL3. Of note, no consensus cleavage site is known for the related presenilins. Instead, it appears that multiple features of the substrate TMD (e.g. helix-destabilizing residues that favor local unwinding of substrates) and TMD-proximal regions determine whether a given protein is cleaved or not [47, 48]. For rhomboid intramembrane serine proteases, with which SPPL3 shares unique mechanistic features, notably the preference to process intact, full-length membrane protein substrates [21, 49], a linear consensus cleavage site/recognition motif was, however, described [50]. Importantly, we were not able to define a SPPL3 consensus cleavage site, yet we showed that merely by replacing its TMD with the MGAT5 TMD, FUT7, a unique fucosyltransferase evidently not undergoing cleavage and secretion [42, 43], is turned into a GTf that is secreted in a manner that exhibits a dependency on SPPL3 strikingly similar to MGAT5, the TMD donor. This demonstrates that in case of a type II membrane protein that is already Golgi-resident (such as FUT7), sequence features present in the TMD alone can dictate intramembrane proteolysis mediated by endogenous SPPL3. Future experiments need to dissect which specific TMD properties determine cleavage by SPPL3 and to what extent SPPL3’s substrate preferences differ from those of other SPP/SPPL family members.

In respect to the mechanism underlying SPPL3-mediated intramembrane proteolysis it is interesting to note that, depending on the SPPL3 substrate, our terminome data contain one (e.g. for MGAT5) or several (e.g. for GALNT2) TMD-derived neo-N-terminal peptides secreted in a SPPL3-dependent manner. The latter is reminiscent of SPPL2a/SPPL2b-mediated substrate processing [41, 51, 52] and could be due to SPPL3-mediated cleavage at multiple sites in a TMD or SPPL3-mediated exopeptidase-like trimming following initial intramembrane cleavage. Alternatively and independently of the intrinsic proteolytic activity of SPPL3, exopeptidases in the secretory pathway or the CM could trim cleavage products. This was previously reported for ST6GAL1 cleaved by BACE1 [53]. In case of trimming, not the most abundant but instead the most N-terminal TMD-derived peptide could be considered the direct SPPL3 cleavage product (e.g. a peptide commencing with Ile19 for GALNT2).

### Physiological function of SPPL3

In higher eukaryotes, the Golgi network harbours a collection of > 100 resident enzymes, which is uniquely enriched in type II membrane proteins, i.e. the majority of Golgi enzymes could theoretically be substrates of SPPL3. Together with the high degree of evolutionary conservation of SPPL3, this suggests that SPPL3-mediated enzyme processing is highly prevalent in the Golgi. This is supported here through the identification of additional Golgi-resident GMEs as SPPL3 substrates (Suppl. Figure 10). Through cleavage, SPPL3 can control the intra-Golgi abundance of GTfs and other GMEs implicated in multiple cellular Golgi glycosylation pathways, which is reflected in the glycosylation changes associated with loss or overexpression of SPPL3 in vitro and in vivo [19, 23–25, 34, 54]. Hence, we postulate that SPPL3 serves the fundamental purpose of maintaining homeostatic levels and distribution of enzymes within the Golgi. It is well documented that Golgi-resident enzymes are asymmetrically distributed in the Golgi and that there are multiple molecular mechanisms in place that ensure proper intra-Golgi distribution and trafficking of Golgi enzymes, mostly through the N-terminal cytosolic tail of GTfs [55, 56]. GOLPH3, for instance, emerged recently as critical factor mediating recycling of GTfs from *trans-* to *medial*-Golgi through interaction with a specific motif within client GTf cytosolic domains, thus maintaining correct intra-Golgi distribution of its client Golgi enzymes [57–59]. By cleaving Golgi GTfs and other GMEs off their membrane anchor, SPPL3 disconnects its substrates from any intra-Golgi localisation signals present in their N-terminus allowing clearance from the Golgi through vesicular trafficking and secretion, thus theoretically opposing the function of e.g. GOLPH3. Acting in tandem, both mechanisms could ensure homeostatic distribution of Golgi enzymes. To test this hypothesis, further in-depth studies interrogating the precise subcellular localization of SPPL3 and the mechanisms governing interactions with its substrates are required. Interestingly, based on the membrane thickness model, intra-Golgi sorting of GTfs is also dictated by the length of their TMDs [56]. Hence, the average TMD length (20 aa) of SPPL3 substrates identified in this study could provide clues for a *medial/trans*-Golgi localisation of SPPL3.

Irrespective of its implications for Golgi biology, our study further strengthens that SPPL3-mediated Golgi enzyme cleavage and subsequent secretion constitutes a source of secreted GTfs present in the extracellular space. First, previous work [19, 20] and this study provide in vitro evidence that loss of SPPL3 leads to depletion of GTfs from CM. Secondly, capitalising on the newly identified SPPL3 cleavage sites, our study also supports that SPPL3-mediated intramembrane proteolysis is a major source of secreted Golgi enzymes in humans in vivo, because we found evidence that several Golgi enzymes present in human blood feature N-termini identical to those generated in a SPPL3-dependent fashion in vitro. Precisely to what extent SPPL3 contributes to the serum levels of Golgi enzymes in vivo will require studies in *SPPL3*-deficient animal models. An important physiological function of SPPL3 may thus also be the generation of extracellular GTfs and GMEs that may be critical for extracellular glycan remodelling processes [9, 11, 12].

Taken together, our study expands the spectrum of SPPL3 substrates, which is instrumental to further dissect the physiological function of SPPL3. In addition, through the identification of TMD-derived neo-N-termini, our study demonstrates that SPPL3-dependent release of type II membrane protein substrates is a direct consequence of intramembrane proteolysis. Finally, we report that substrate TMDs can determine cleavage by SPPL3, opening up avenues for further studies dissecting molecular mechanisms governing interactions of SPPL3 and its substrates, and we present evidence that SPPL3 cleavage products are present in human serum.

## Supplementary Information

Below is the link to the electronic supplementary material.

Supplementary file1 (PDF 6601 KB)

Supplementary file2 (XLSX 8224 KB)

## Data Availability

Proteome data have been deposited to the ProteomeXchange Consortium via the PRIDE partner repository [1] with the dataset identifier PXD028769. Materials generated in the course of this study will be made available upon reasonable request to the corresponding author (MV).

## Abbreviations

aa: Amino acid
ACN: Acetonitrile
BCA: Bicinchoninic acid assay
BSA: Bovine serum albumin
CBB: Coomassie Brilliant Blue G250
cDNA: Complementary DNA
CDS: Coding DNA sequence
CM: Conditioned medium
CMW: Chloroform/methanol/water
crRNA: CRISPR RNA
Dkk1: Dickkopf1
DTT: Dithiothreitol
EDTA: Ethylenediaminetetraacetic acid
ER: Endoplasmic reticulum
FA: Formaldehyde
FACS: Fluorescence-activated cell sorting
FAIMS: High-field asymmetric-waveform ion-mobility spectrometry
FDR: False discovery rate
gDNA: Genomic DNA
GME: Glycan-modifying enzyme
GTf: Glycosyltransferase
HRP: Horseradish peroxidase
HYTANE: Hydrophobic tagging-assisted N-termini enrichment
KO: Knock-out
LC–MS: Liquid chromatography–mass spectrometry
LRP: LDL-receptor-related protein
mAb: Monoclonal antibody
MCS: Multiple cloning site
MS: Mass spectrometry
NCE: Normalized collision energies
NK: Natural killer
pAb: Polyclonal antibody
PBS: Phosphate buffered saline
PCR: Polymerase chain reaction
PNS: Post-nuclear supernatant
PSSM: Position-specific scoring matrix
PVDF: Polyvinylidene fluoride
RIPA: Radioimmunoprecipitation assay buffer
RNP: Ribonucleoprotein
SDS: Sodium dodecyl sulfate
sgRNA: Single guide RNA
ssODN: Single-stranded oligodeoxynucleotides
TCA: Trichloroacetic acid
TCEP: Tris(2-carboxyethyl)phosphine hydrochloride
TEAB: Triethylammonium bicarbonate
TFA: Trifluoroacetic acid
TMD: Transmembrane domain
TMT: Tandem mass tag
tracrRNA: Trans-activating CRISPR RNA
WGA: Wheat germ agglutinin
WT: Wild-type

## Code availability

R and Python code used for data analysis is available upon request.
