# Supplementary material for: N-terminome analyses underscore the prevalence of SPPL3-mediated intramembrane proteolysis among Golgi-resident enzymes and its role in Golgi enzyme secretion
Source: Cell Mol Life Sci. 2022 Mar 13;79(3):185. doi: 10.1007/s00018-022-04163-y (PMC8918473; doi:10.1007/s00018-022-04163-y)
Supplement: Supplementary file 1 — Supplementary file1 (PDF 6601 KB) [file 18_2022_4163_MOESM1_ESM.pdf]

Supplementary Figures

N-terminome analyses underscore the prevalence of SPPL3-mediated intramembrane proteolysis among Golgi-resident enzymes and its role in Golgi enzyme secretion

Laura Hobohm[1], Tomas Koudelka[2], Fenja H. Bahr[1], Jule Truberg[1], Sebastian Kapell[3], Sarah-Sophie Schacht[1], Daniel Meisinger[1], Marion Mengel[1], Alexander Jochimsen[1], Anna Hofmann[1], Lukas Heintz[1], Andreas Tholey[2], and Matthias Voss[1,*]

[1] Institute of Biochemistry, Kiel University, Rudolf-Höber-Str. 1, 24118 Kiel, Germany

[2] Systematic Proteome Research & Bioanalytics, Institute for Experimental Medicine, Kiel University, 24105 Kiel, Germany.

[3] National Bioinformatics Infrastructure Sweden (NBIS), Science for Life Laboratory, Department of Biochemistry and Biophysics, Stockholm University, 10691 Stockholm, Sweden.

* To whom correspondence should be addressed: mvoss@biochem.uni-kiel.de

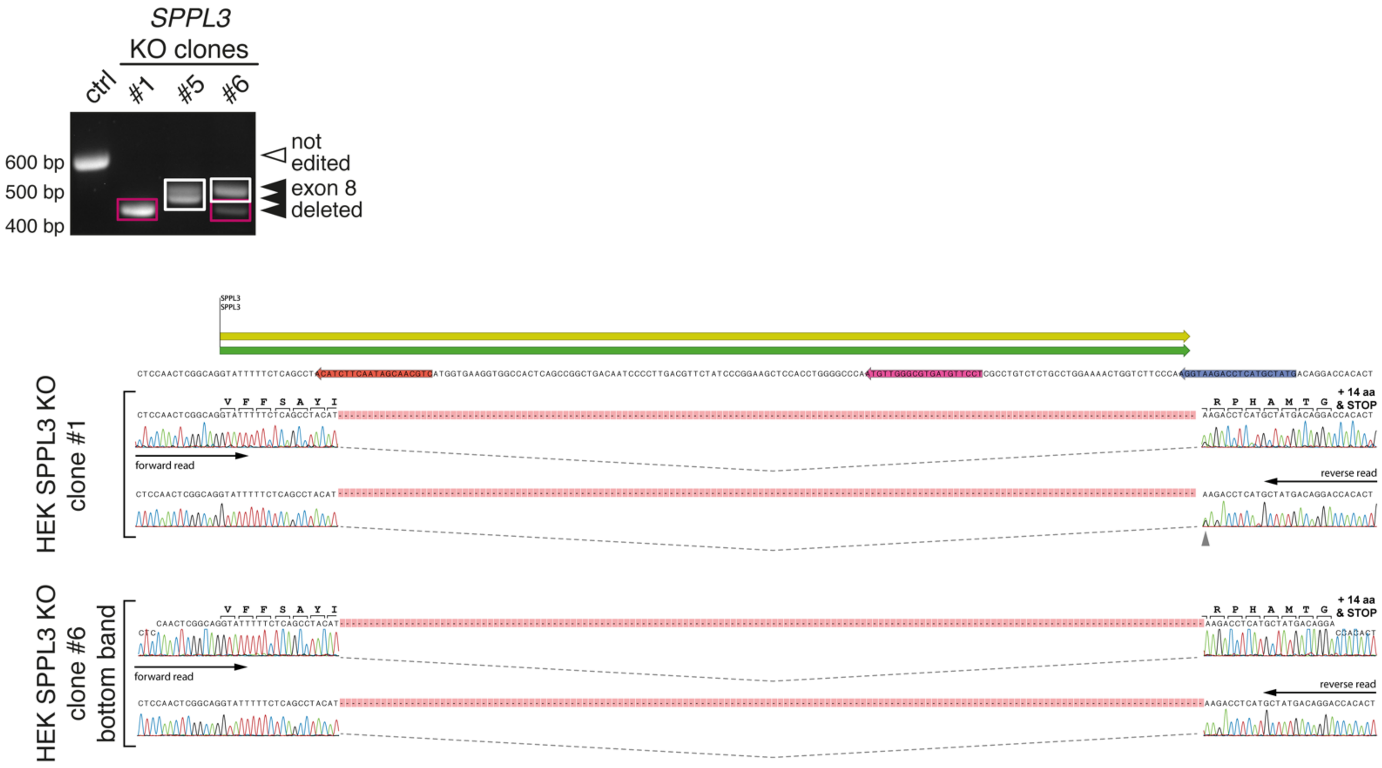

Suppl. Fig. 1

Suppl. Fig. 1: Sanger sequencing confirming editing of the *SPPL3* locus in HEK293 cells. Only truncated PCR products were amplified from gDNA of edited HEK293 clones #1, #5 and #6 (top panel, which is identical to the one shown in Fig. 1b) demonstrating that only edited alleles are present in these cells. Individual PCR products were gel-purified, Sanger sequenced and aligned to the human reference genome (top) confirming deletions between two sgRNA binding sites (colour-coded arrows). Note that clean assembly of Sanger reads was only possible for PCR products highlighted in magenta boxes, as additional bands in clone #5 and #6 (white boxes) displayed only marginal difference in length (confirmed by capillary gel electrophoresis, data not shown) and thus could not be separated sufficiently from each other. Grey arrowheads indicate indel variants occurring in addition to the larger deletions. Putative translation products of each read are shown above each assembled sequence starting with the first full codon in exon 8 (corresponding to Val204 in SPPL3 (Uniprot Q8TCT6-2)).

(next page)

Suppl. Fig. 2: Sanger sequencing of predicted sgRNA off-target binding sites in HEK293 cells. Highly ranked putative off-target binding sites are listed for each of the three sgRNAs used (as predicted by IDT's online tool (Coralville, USA) and CHOPCHOP [1]). Primers specifically amplifying a < 1,000 bp fragment comprising the predicted off-target site were designed using Primer-BLAST. PCR was performed on gDNA isolated from the generated *SPPL3*-deficient HEK293 clones #1, #5 and #6. PCR products were gel-purified and Sanger sequenced. Clipped reads comprising the putative off-target binding site (indicated by arrows), a potential 3' PAM as well as 10 nucleotides upstream and downstream are shown.

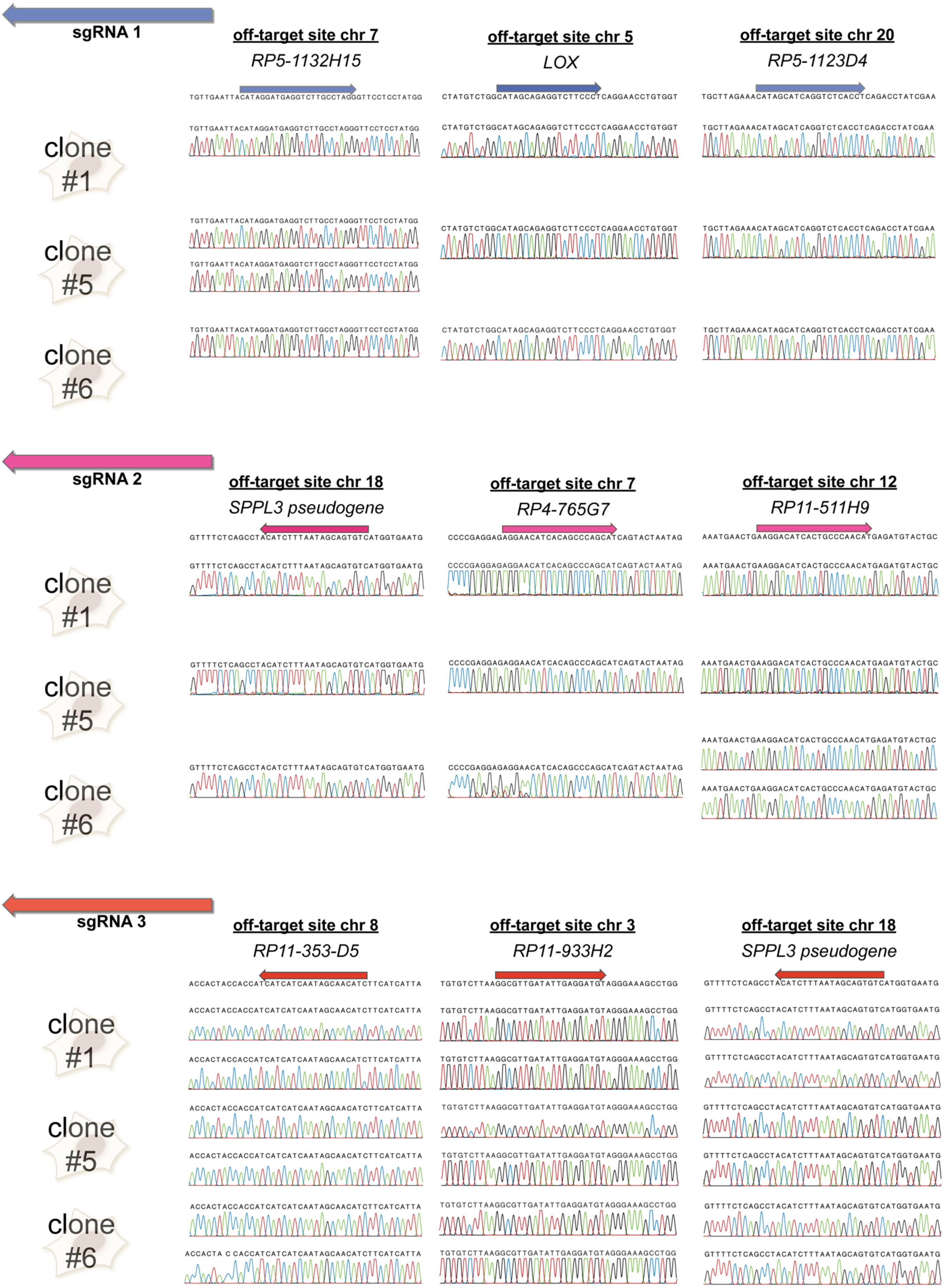
sgRNA 1
off-target site chr 7
RP5-1132H15
off-target site chr 5
LOX
off-target site chr 20
RP5-1123D4
clone #1
clone #5
clone #6
sgRNA 2
off-target site chr 18
SPPL3 pseudogene
off-target site chr 7
RP4-765G7
off-target site chr 12
RP11-511H9
clone #1
clone #5
clone #6
sgRNA 3
off-target site chr 8
RP11-353-D5
off-target site chr 3
RP11-933H2
off-target site chr 18
SPPL3 pseudogene
clone #1
clone #5
clone #6

Suppl. Fig. 2

(next page)

Suppl. Fig. 3: Identification of substrates of endogenous SPPL3 in HEK293 cells. (**a**) Pre-HYTANE analysis of HEK293 CM. Prior to HYTANE, TMT-labelled tryptic peptides were analysed and quantified. In total, 1,467 proteins were quantified. Data labelling/colouring and thresholds are set as in Fig. 2b. (**b**) 1D-LC FAIMS data highlighting the efficiency of the HYTANE strategy to deplete internal trypsin (Arg-C) generated peptides. Number of internal (*left*) and N-terminal (*right*) peptides (either acetylated of TMT-labelled) detected before enrichment ("Pre-HYTANE") and after the depletion of internal tryptic (Arg-C) peptides using the HYTANE strategy ("HYTANE"). Prior to depletion, N-termini are rarely chosen for MS/MS fragmentation, which become the majority with ≈90% of detected peptides following HYTANE. (**c**) Data from Fig. 2c with enlarged area covering type II membrane protein-derived peptides (filtered for intramembrane origin with x = 30 aa, see Fig. 2e) significantly ($p < 0.05$) more abundant in CM of parental HEK293 cells, but with a log2-fold change in the indicated range below the median ± 2SD cut-off applied throughout Fig. 2. Large black dots depict N-terminal peptide derived from proteins previously found to be altered upon changes in SPPL3 expression in secretome data [2]. Coloured dots denote peptides from the same type II protein. (**d**) Mapping of cleavage sites identified for substrates denoted by a black dot in (**c**). (**e**) *(left)* Post-nuclear supernatants of cell homogenates were loaded on a discontinuous sucrose gradient as depicted and separated by centrifugation. Vesicle fractions (F1 to F3) at the interphases were collected, sedimented at 100,000 g and lysed. *(right)* Immunoblot analysis of isolated fractions revealed marked enrichment of Golgi markers and SPPL3 in F1 (*bottom half*), while no other subcellular markers analysed were enriched in F1. mem, lysates of carbonate-washed membranes; WCL, whole cell lysate (RIPA). (**f**) Volcano plot depicting proteins identified by label-free quantification of F1 characterized in (**e**) and isolated from three *SPPL3* KO HEK293 clones and three replicates of parental HEK293 (ctrl) cells. 4,056 proteins were detected and 110 proteins had their values imputed. Type II membrane proteins are shown in black, unless significantly more abundant in *SPPL3* KO or parental cells (red and blue, respectively). Thresholds are indicated by red dashed lines at $p = 0.05$ and a log2-fold change of Median ± 2 SD.

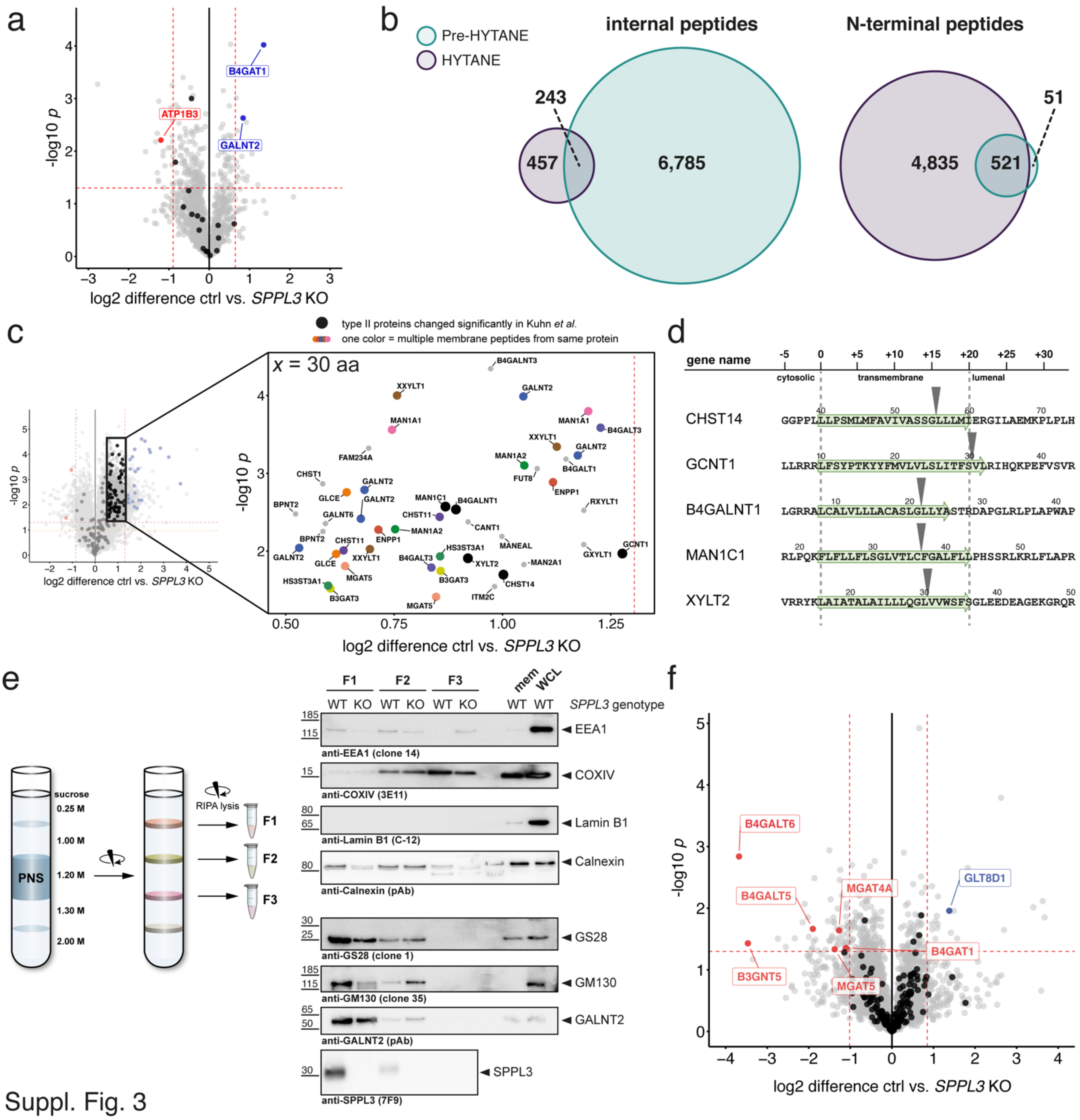
a
ATP1B3
B4GAT1
GALNT2
-log10 p
log2 difference ctrl vs. SPPL3 KO
b
Pre-HYTANE
HYTANE
internal peptides
N-terminal peptides
243
457
6,785
4,835
521
51
c
type II proteins changed significantly in Kuhn et al.
one color = multiple membrane peptides from same protein
x = 30 aa
d
gene name
cytosolic
transmembrane
lumenal
CHST14
GCNT1
B4GALNT1
MAN1C1
XYLT2
e
sucrose
PNS
RIPA lysis
F1
F2
F3
SPPL3 genotype
EEA1
COXIV
Lamin B1
Calnexin
GS28
GM130
GALNT2
SPPL3
anti-EEA1 (clone 14)
anti-COXIV (3E11)
anti-Lamin B1 (C-12)
anti-Calnexin (pAb)
anti-GS28 (clone 1)
anti-GM130 (clone 35)
anti-GALNT2 (pAb)
anti-SPPL3 (7F9)
f
B4GALT6
B4GALT5
MGAT4A
GLT8D1
B4GAT1
B3GNT5
MGAT5

Suppl. Fig. 3

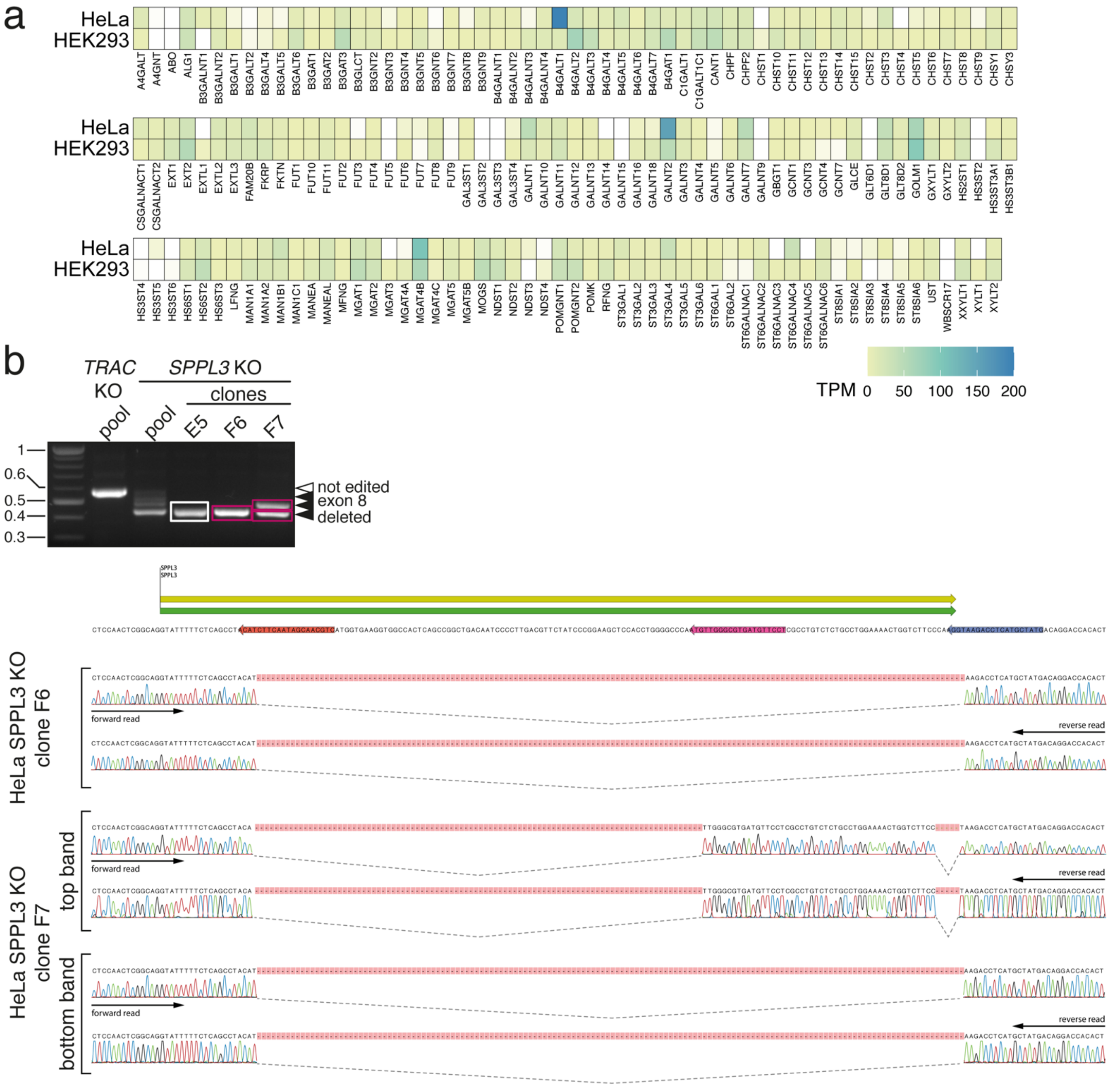

Suppl. Fig. 4

Suppl. Fig. 4: Generation of *SPPL3*-deficient HeLa cells. (**a**) Transcript levels of genes encoding potential SPPL3 substrates in HeLa and HEK293 cells. A heatmap was generated from published transcriptome data [3]. Data were filtered for genes encoding type II membrane proteins implicated in Golgi glycosylation pathways (based on [4, 5]) and previously identified SPPL3 substrates. (**b**) Sanger sequencing confirming editing of the *SPPL3* locus in HeLa cells. Only truncated PCR products were amplified from gDNA of edited HeLa clones (top panel, which is identical to the one shown in Fig. 3A) demonstrating that only edited alleles are present in these cells. Individual PCR products were gel-purified, Sanger sequenced and aligned to the human reference genome (top) confirming deletions between two sgRNA binding sites (colour-coded arrows). Note that clean assembly of Sanger reads was only possible for PCR products highlighted in magenta boxes, as additional bands in clone E5 (white box) displayed only marginal difference in length (confirmed by capillary gel electrophoresis, data not shown) and thus precluded clean Sanger sequencing.

(next page)

Suppl. Fig. 5: Sanger sequencing of predicted sgRNA off-target binding sites in HeLa cells. For details, see Suppl. Fig. 2 legend.

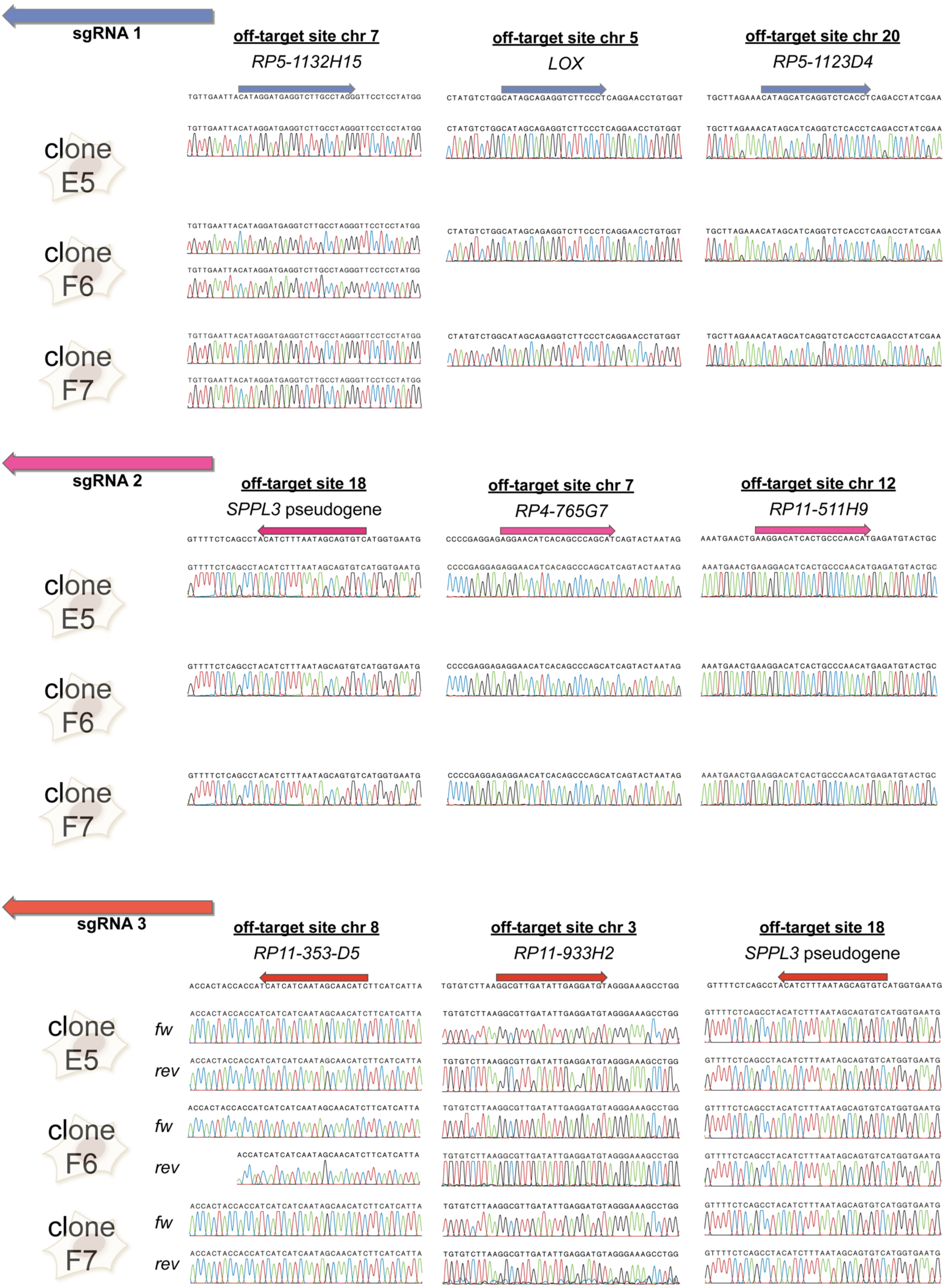

sgRNA 1
off-target site chr 7
RP5-1132H15
TGTTGAATTACATAGGATGAGGTCTTGCCTAGGGTTCCTCCTATGG
off-target site chr 5
LOX
CTATGTCTGGCATAGCAGAGGTCTTCCCTCAGGAACCTGTGGT
off-target site chr 20
RP5-1123D4
TGCTTAGAAACATAGCATCAGGTCTCACCTCAGACCTATCGAA
clone E5
clone F6
clone F7
sgRNA 2
off-target site 18
SPPL3 pseudogene
GTTTTCTCAGCCTACATCTTTAATAGCAGTGTCATGGTGAATG
off-target site chr 7
RP4-765G7
CCCCGAGGAGAGGAACATCACAGCCCAGCATCAGTACTAATAG
off-target site chr 12
RP11-511H9
AAATGAACTGAAGGACATCACTGCCCAACATGAGATGTACTGC
clone E5
clone F6
clone F7
sgRNA 3
off-target site chr 8
RP11-353-D5
ACCACTACCACCATCATCATCAATAGCAACATCTTCATCATTA
off-target site chr 3
RP11-933H2
TGTGTCTTAAGGCGTTGATATTGAGGATGTAGGGAAAGCCTGG
off-target site 18
SPPL3 pseudogene
GTTTTCTCAGCCTACATCTTTAATAGCAGTGTCATGGTGAATG
clone E5
fw
rev
clone F6
fw
rev
clone F7
fw
rev

Suppl. Fig. 5

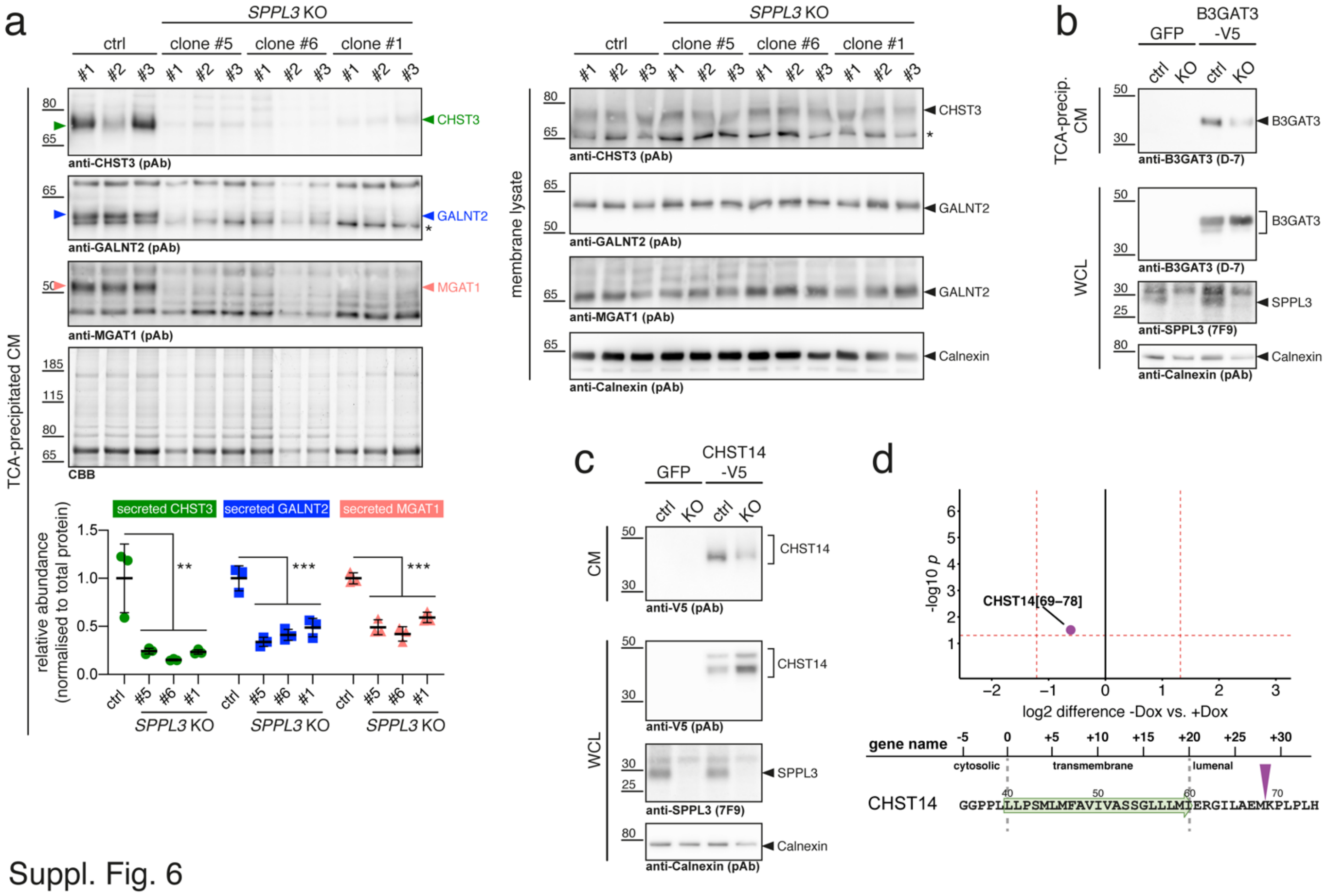

Suppl. Fig. 6

Suppl. Fig. 6: Immunoblot validation of novel SPPL3 substrates in *SPPL3*-deficient HEK293 cells. (**a**) Quantitative analysis of SPPL3-dependent secretion of CHST3, GALNT2 and MGAT1 in HEK293 *SPPL3* KO cells. CHST3, GALNT2 and MGAT1 levels in TCA-precipitated CM *(top left)* and lysates of carbonate-washed membranes *(top right)* were analysed in parental HEK293 cells (ctrl) as well as three HEK293 *SPPL3* KO clones (each in three replicates, #1 to #3). The relative abundance of secreted CHST3, GALNT2 and MGAT1 in TCA-precipitated CM of SPPL3 KO cells compared to parental cells was calculated and plotted *(bottom right)* following densitometric immunoblot quantification and normalisation to total protein content as assessed by CBB staining. Plotted data include individual data points for the three replicates as well as mean ± SD. *, unspecific band (not quantified). **, $p < 0.01$, ***, $p < 0.001$ (one-way ANOVA). (**b**) Validation of B3GAT3 as SPPL3 substrate. TCA-precipitated CM and whole-cell lysates (WCL) were prepared from HEK293 cells and *SPPL3*-deficient HEK293 cells (clone #1) transfected with GFP or C-terminally V5-tagged B3GAT3. (**c**) Validation of CHST14 as SPPL3 substrate, conducted as detailed in (**b**), but cells transfected with GFP or C-terminally V5-tagged CHST14. (**d**) SPPL3-independent generation of a CHST14 neo-N-terminal peptide. (*top*) Volcano plot from Fig. 4d was modified to only show CHST14-derived peptides and only one peptide comprising aa 69 to 78 of CHST14 was detected in the dataset. (*bottom*) The identification of CHST14[69-78] suggests that SPPL3-independent cleavage of CHST14 can occur in the membrane-proximal luminal stem yet outside of the TMD.

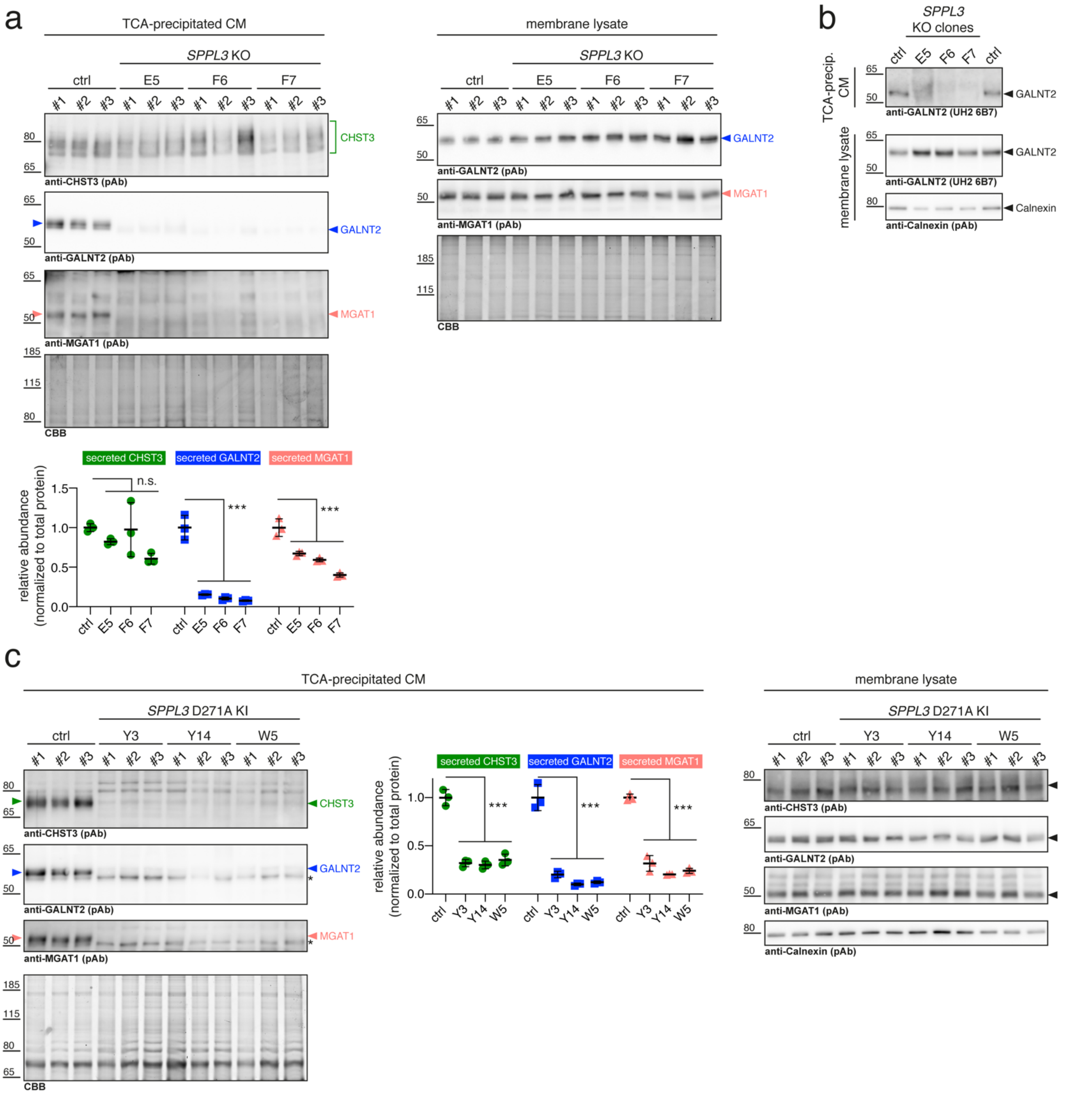

Suppl. Fig. 7

Suppl. Fig. 7: Immunoblot validation of novel SPPL3 substrates in SPPL3-deficient HeLa and HEK293 SPPL3 D271A knock-in cells. (**a**) Quantitative analysis of SPPL3-dependent secretion of CHST3, GALNT2 and MGAT1 in *SPPL3* KO HeLa cells. CHST3, GALNT2 and MGAT1 levels in TCA-precipitated CM *(left)* and lysates of carbonate-washed membranes *(right)* were analysed in parental HEK293 cells (ctrl) as well as three HeLa KO clones (each in three replicates, #1 to #3). The relative abundance of secreted CHST3, GALNT2 and MGAT1 in TCA-precipitated CM from KO cells compared to parental cells was calculated and plotted *(bottom left panel)* following densitometric immunoblot quantification and normalisation to total protein content as assessed by CBB staining. Plotted data include individual data points for the three replicates as well as mean ± SD. (**b**) Immunoblot analysis of GALNT2 secretion from HeLa cells as shown in Fig. 5b but with a different GALNT2-specific antibody. (**c**) Quantitative analysis of SPPL3-dependent secretion of CHST3, GALNT2 and MGAT1 in HEK293 SPPL3 D271 knock-in cells. CHST3, GALNT2 and MGAT1 levels in TCA-precipitated CM *(left)* and lysates of carbonate-washed membranes *(right)* were analysed in parental HEK293 cells (ctrl) as well as three HEK293 SPPL3 D271A knock-in clones (each in three replicates, #1 to #3). The relative abundance of secreted CHST3, GALNT2

and MGAT1 in TCA-precipitated CM from SPPL3 D281A knock-in cells compared to parental cells was calculated and plotted *(centre panel)* following densitometric immunoblot quantification and normalisation to total protein content as assessed by CBB staining. Plotted data include individual data points for the three replicates as well as mean ± SD. *, unspecific band (not quantified). ***, $p < 0.001$ (one-way ANOVA). n.s., not significant.

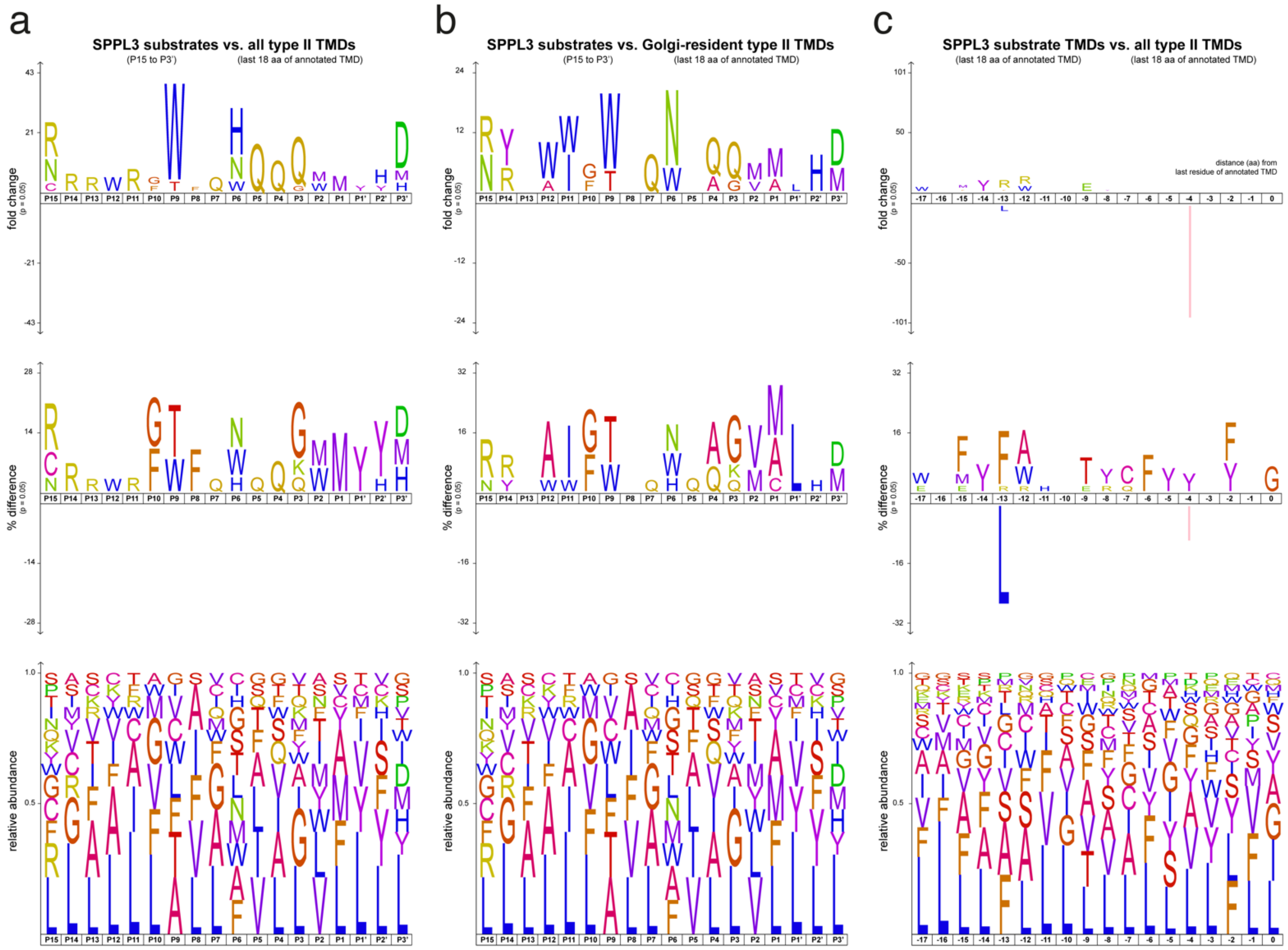

Suppl. Fig. 8

Suppl. Fig. 8: IceLogo analyses of SPPL3 substrates. (**a**, **b**) IceLogo analyses of regions flanking SPPL3 cleavage sites (P15 to P3'). Peptide sequences used for the analysis are detailed in Suppl. Tab. S10 and are identical to the ones used for Fig. 6b. The reference dataset consisted of the last 18 aa of the annotated TMDs of all (**a**) or only Golgi-localised (**b**) type II membrane proteins. Aa enrichment and depletion was calculated using the fold change (top panels) and the % difference setting (middle panels). Note that at $p = 0.05$ no de-enrichment of aa residues in region analysed was noted. The relative abundance of individual aa at specific positions is also plotted (bottom panel). Residue colouring is based on IceLogo's hydrophobicity scale (hydrophobic residues in blue). (**c**) IceLogo analysis of the last 18 TMD residues of all confidently identified SPPL3 substrates (see Suppl. Fig. 10). The reference dataset included the corresponding region of all type II proteins, yet excluding proteins in the input dataset. All input and reference data are listed in Suppl. Tab. S10.

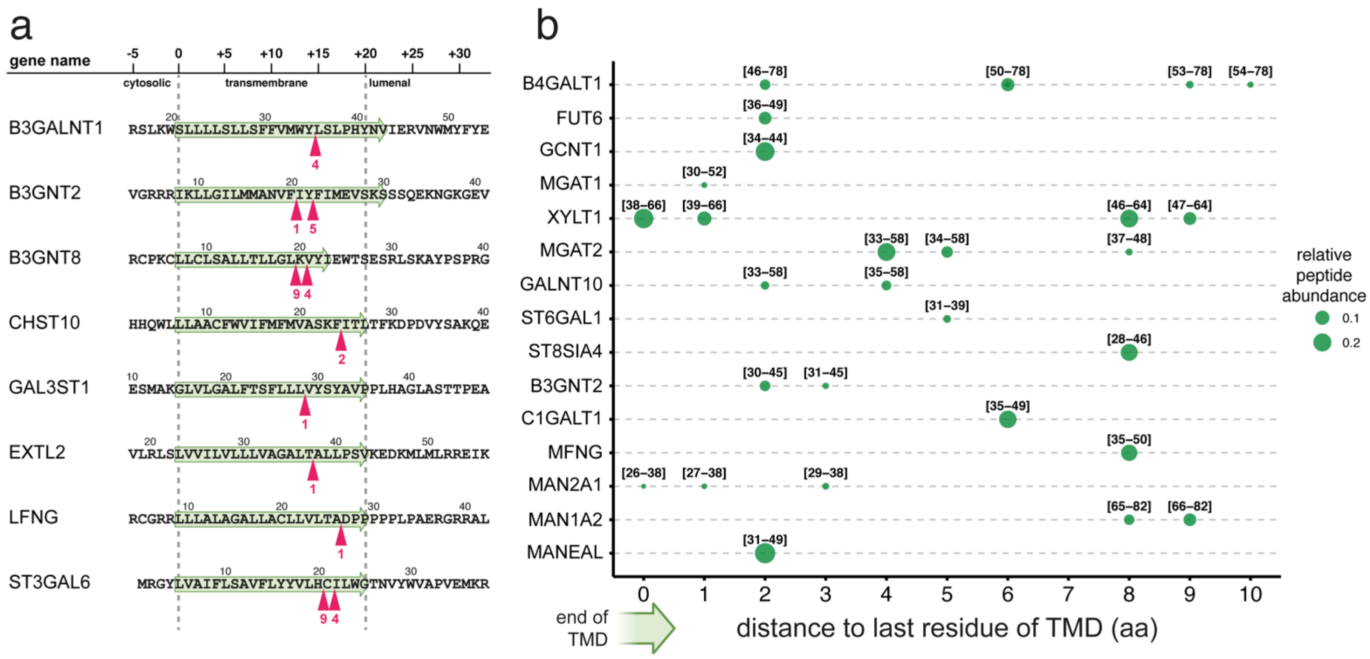

Suppl. Fig. 9

Suppl. Fig. 9: Potential Golgi GME-derived intramembrane cleavage products and membrane proximal peptides found in human plasma. (**a**) Semi-tryptic peptides present in the *Human Plasma PeptideAtlas* (Build 2021-07) that map to the transmembrane domain of Golgi GMEs displaying a type II membrane protein topology and not yet conclusively identified as SPPL3 substrates. Arrows indicate intramembrane cleavage events that would give rise to such neo-N-termini. Numbers under the arrow indicate "times observed" in the database. (**b**) Semi-tryptic and tryptic peptides for type II membrane protein Golgi GMEs found in *Human Plasma PeptideAtlas* that map to the first 10 luminal aa acids (position = last residue of annotated TMD). Relative peptide abundance was calculated for each parent protein by dividing the number of observations for each peptide by the total observations made for any peptide deriving from the parent protein. Numbers in brackets indicate peptide positions.

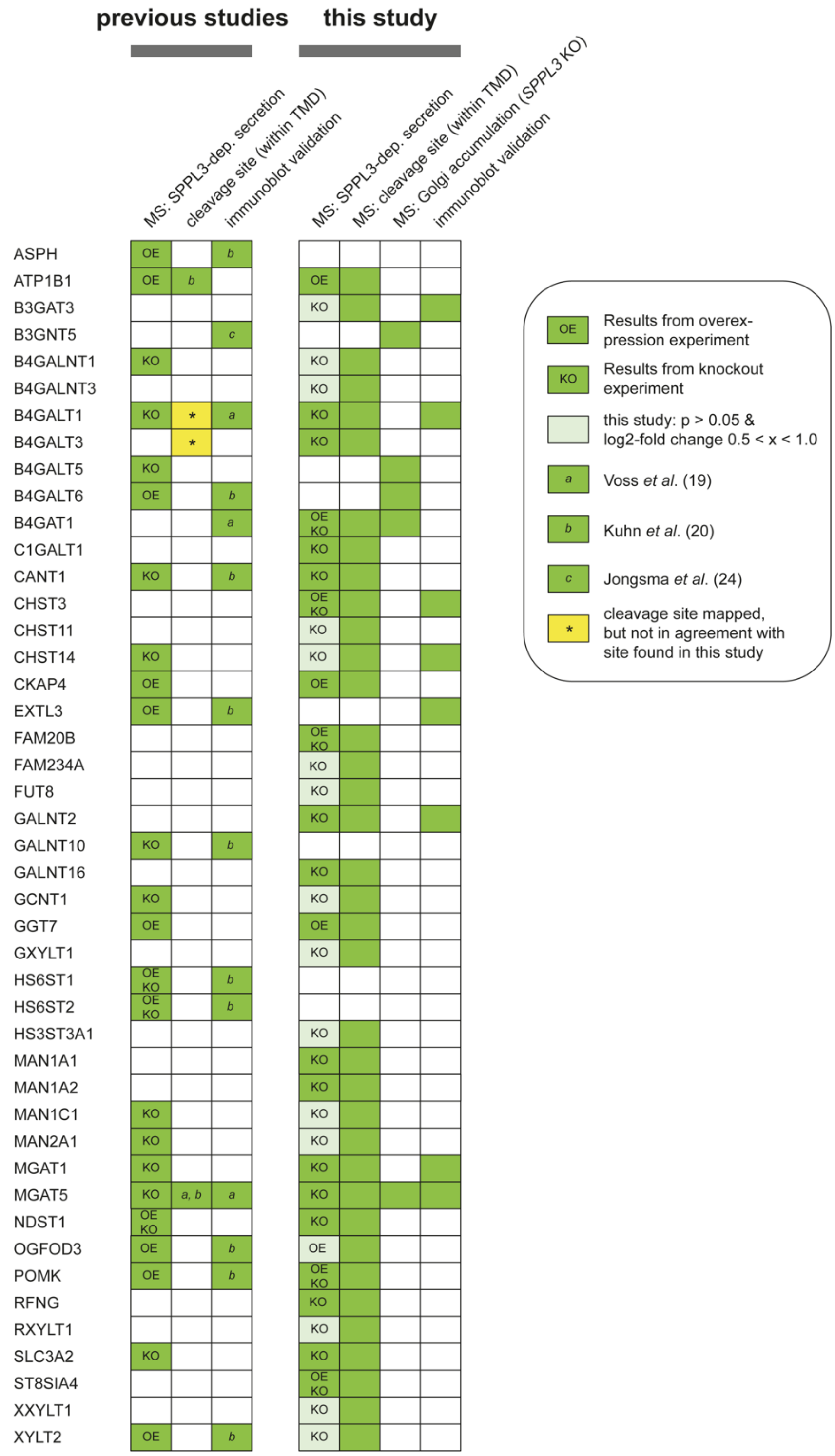

Suppl. Fig. 10

Suppl. Fig. 10: Summary of the SPPL3 substrates identified in this study and previous studies. For all substrates, given here by gene name and listed in alphabetical order, this figure summarises evidence presented in this study (*right*) and in previous studies (*left*) that support their SPPL3-mediated proteolytic processing. Note that, for the purpose of this simplified overview, evidence for both murine and human experimental models is considered and summarized under the gene name of the human substrate orthologue. For B4GALT1 and B4GALT3 the previously reported cleavage sites Tyr41|Leu42 and Met24|Tyr25, respectively (which were based on the identification of semi-tryptic peptides originating in the respective TMDs), are not in agreement with the cleavage sites determined here (Fig. 2h & 3e). To be considered in this table, a given SPPL3 substrate needs to fulfil at least two of the criteria leading to the omission of several candidate SPPL3 substrates identified to be secreted in a SPPL3-dependent fashion following secretome enrichment by Kuhn *et al*. [2].

References

[1] Labun K, Montague TG, Gagnon JA, et al (2016) CHOPCHOP v2: a web tool for the next generation of CRISPR genome engineering. Nucleic Acids Res 44:W272–W276. https://doi.org/10.1093/nar/gkw398

[2] Kuhn P-H, Voss M, Haug-Kröper M, et al (2015) Secretome Analysis Identifies Novel Signal Peptide Peptidase-Like 3 (SPPL3) Substrates and Reveals a Role of SPPL3 in Multiple Golgi Glycosylation Pathways. Mol Cell Proteomics 14:1584–1598. https://doi.org/10.1074/mcp.M115.048298

[3] Thul PJ, Åkesson L, Wiking M, et al (2017) A subcellular map of the human proteome. Science 356:eaal3321. https://doi.org/10.1126/science.aal3321

[4] Schjoldager KT, Narimatsu Y, Joshi HJ, Clausen H (2020) Global view of human protein glycosylation pathways and functions. Nat Rev Mol Cell Biol 21:729–749. https://doi.org/10.1038/s41580-020-00294-x

[5] Moremen KW, Ramiah A, Stuart M, et al (2018) Expression system for structural and functional studies of human glycosylation enzymes. Nat Chem Biol 14:156–162. https://doi.org/10.1038/nchembio.2539
